# Seagrass and macrophyte mediated CO_2_ and CH_4_ dynamics in shallow coastal waters

**DOI:** 10.1371/journal.pone.0203922

**Published:** 2018-10-08

**Authors:** Kakolee Banerjee, A. Paneerselvam, Purvaja Ramachandran, Dipnarayan Ganguly, Gurmeet Singh, R. Ramesh

**Affiliations:** National Centre for Sustainable Coastal Management (NCSCM), Ministry of Environment, Forest and Climate Change (Government of India), Anna University Campus, Chennai, Tamil Nadu, India; Universidade de Aveiro, PORTUGAL

## Abstract

Seagrass meadows are among the most important coastal/ marine ecosystems for long-term carbon storage and conditioning of coastal waters. A combined air-water flux of CO_2_ and CH_4_ from the seagrass meadows was studied for the first time from Asia’s largest brackish–water lagoon, Chilika, India. Ecosystem-based comparisons were carried out during two hydrologically different conditions of dry and wet seasons in the seagrass dominated southern sector (SS); macrophyte-dominated northern sector (NS); the largely un-vegetated central sector (CS) and the tidally active outer channel (OC) of the lagoon. The mean fluxes of CO_2_ from SS, NS, CS and OC were 9.8, 146.6, 48.4 and 33.0mM m^-2^d^-1^, and that of CH_4_ were 0.12, 0.11, 0.05 and 0.07mM m^-2^d^-1^, respectively. The net emissions (in terms of CO_2_ equivalents), considering the global warming potential of CO_2_ (GWP: 1) and CH_4_ (GWP: 28) from seagrass meadows were over 14 times lower compared to the macrophyte-dominated sector of the lagoon. Contrasting emissivity characteristics of CO_2_ and CH_4_ were observed between macrophytes and seagrass, with the former being a persistent source of CO_2_. It is inferred that although seagrass meadows act as a weak source of CH_4_, they could be effective sinks of CO_2_ if land-based pollution sources are minimized.

## Introduction

Global average atmospheric carbon dioxide (CO_2_) and methane (CH_4_) concentrations have reached 404 parts per million (ppm; [1]) and 1834 parts per billion (ppb; [2]), respectively, which are more than 43% and 154% higher than the pre-industrial concentrations. In addition to the emission reduction strategies, biological sequestration of atmospheric greenhouse gases (GHGs) is considered a primary mechanism to mitigate global climate change. Vegetated coastal ecosystems, such as mangroves, seagrass meadows, and salt marshes effectively sequester CO_2_, and can store it on millennial time scales [3–5]. The seagrass ecosystem occupies 0.1–0.2% of the coastal oceans, which is an estimated global coverage of 3.45 x 10^5^ km^2^ [6]. Seagrass plays a large role by supplying food [7], providing breeding and nurseries [8], reducing hydrodynamic stress [9], increasing pH through CO_2_ and nutrient uptake [10, 11]. Seagrass ecosystems are well known for their significant but largely variable carbon (C) storage potential [12]; as evident from their high contribution (10–18%) to the total oceanic carbon burial despite covering less than 0.1% of the total ocean floor [13, 14].

Changing climate and increase in anthropogenic stress could substantially alter the natural processes of coastal ecosystems, from being net sinks of C to sources [13–15]. Similarly, C assimilation through photosynthesis, and heterotrophic mineralization of assimilated C in coastal ecosystems, largely depends on several environmental factors under oxic or anoxic conditions. Methanogenesis is the ultimate pathway to mineralize organic compounds present in natural wetlands, and the principal end-product of anaerobic mineralization processes is methane [16]. Natural wetlands are considered to be single largest (~30%) contributor of the global methane emission [17].

Fourqurean et al [18] and Pendleton et al [19] argued that the deterioration of salt marshes, mangrove and seagrass beds that serve as carbon sinks may contribute to climate change through re-emissions of locked carbon dioxide and other GHGs. Globally, intensive research was undertaken in determining the source-sink assessment of GHG emissions from mangrove waters [20–22] and estuaries [23–25], whereas very few studies have been reported from seagrass meadows globally [26], even less so from the seagrass meadows of India. Historically, the role of seagrass meadows in combating climate change through carbon sequestration and storage had been virtually ignored in global carbon budgets [13] but is however recognized in recent times. Significant lack of studies on GHG fluxes from seagrass meadows compared to other blue carbon ecosystems is probably due to the complexities associated with sampling in such submerged habitats. This knowledge gap on the GHG fluxes from seagrass ecosystems limits our capacity to formulate strategies to mitigate climate change, based on their carbon sink potentials. It is therefore essential to understand and accurately account for the factors regulating GHG fluxes in seagrass ecosystems [27]. A better understanding of GHG flux dynamics from the seagrass meadows could further justify the need for conservation and restoration and their significant role in the climate change mitigation.

**Fig 1:**
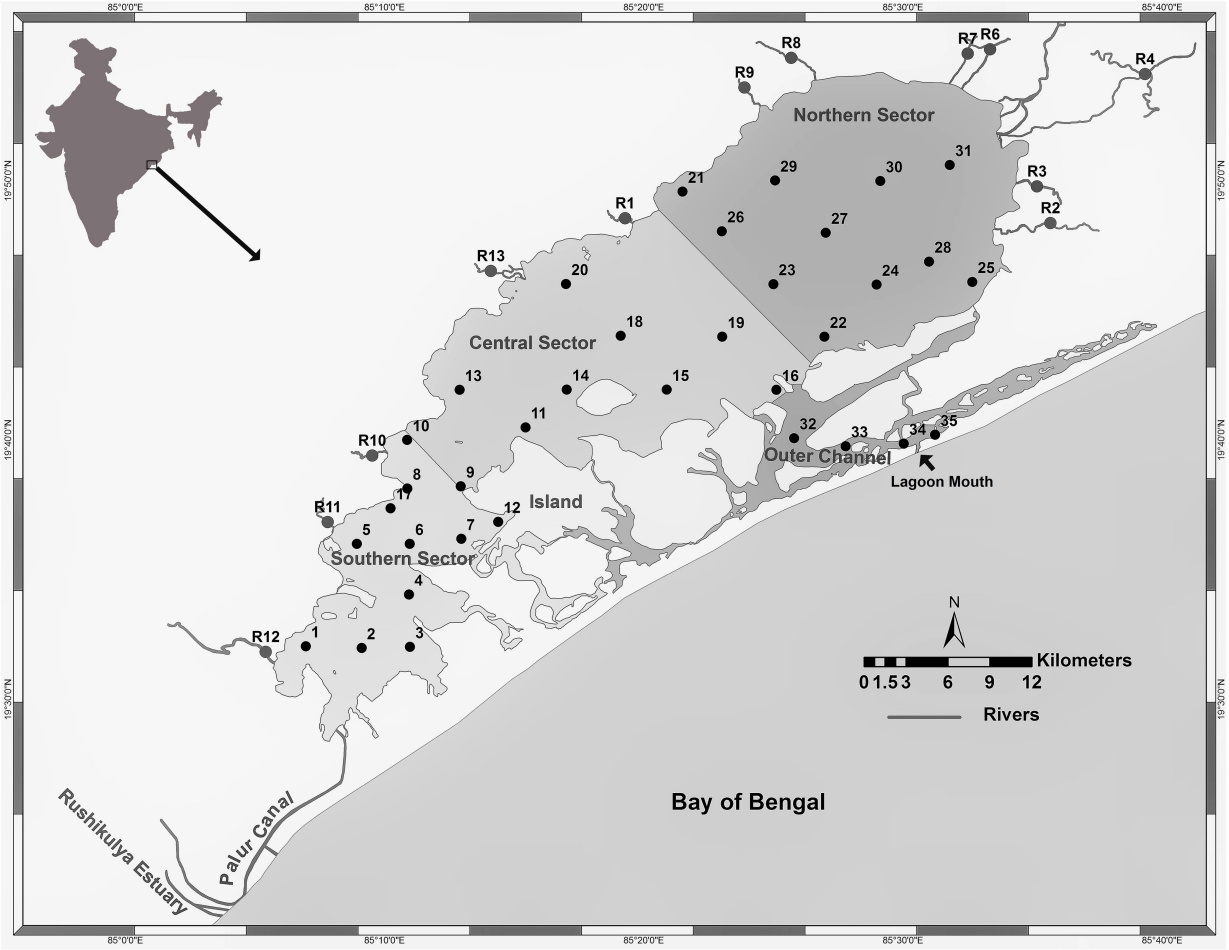
Map of Chilika Lagoon with sectoral zonation; i) Northern Sector (macrophyte dominated), ii) Central Sector (partially macro algae-SAV dominated), iii) Southern Sector (seagrass dominated), and iv) Outer Channel (tidally active). ArcGIS ver 10.5.1 was used for creating the map.

In India, seagrass meadows are distributed in various pockets along the east and west coasts, with a total area of ~517 km^2^ [28]. There have been a few studies focused on seagrass productivity [29, 30] from Indian coastal waters. This study is the first in determining the air-water fluxes of key GHGs (CO_2_ and CH_4_) from a seagrass meadow in India. Additionally, efforts have been made to identify the factors that regulate the GHG flux dynamics from the heterogeneous coastal lagoon characterized by variable salinity with respect to both space and time. The present work was undertaken in Chilika Lagoon (Fig 1), where dense seagrass meadows and other aquatic macrophytes exist in the lagoon. Fluxes of CO_2_ and CH_4_ were studied throughout the lagoon to highlight the role of seagrass in emissivity characteristics.

## Material and methods

### Study area

Chilika Lagoon is a pear shaped coastal lagoon, located on the east coast of India with a unique assemblage of marine, brackish and fresh water ecosystems (Fig 1; Lat. 19°28' - 19°54'N; Long. 85°05'- 85°38'E). It is the largest brackish water lagoon in Asia, known for its rich floral and faunal biodiversity, and was the first in India to be designated as a wetland of international importance under the Ramsar Convention (site# 229). This shallow water lagoon with an average depth of 0.9–2.6 m, spreads over an area of ~906 km^2^ during pre-monsoon (March-June), which swells up to ~1,020 km^2^with average depth between 1.8 and 3.7 m during the monsoon (July-October) [31]. The lagoon dynamics are primarily controlled by fifty-two rivers/rivulets that are spatially distributed along its west and north periphery, delivering significant volume of freshwater, nutrients and suspended matter into the lagoon during the monsoon [32]. The lagoon is classified into four sectors; 1) Northern sector (NS) receiving the highest river discharge, 2) Central Sector (CS), the mixing zone of all sectors, 3) Southern Sector (SS) connected with the sea through Palur Canal and 4) Outer Channel (OC) connected to the Bay of Bengal. During monsoon ~ 90% riverine input to the lagoon is through the northern sector and the remaining ~ 7% and ~ 3% inputs are through the central and southern sectors [33] respectively. The water depth among the sectors follows the order: Outer Channel > Southern Sector > Central Sector > Northern Sector [33].

The freshwater regime and high nutrient level support healthy growth of submerged macrophytes such as *Stuckenia pectinata (Potamogeton)*, *Chara*, and *Hydrilla* in the Northern Sector throughout the year [34]. *Stuckenia pectinata* (locally known as Charidal), which can tolerate wide salinity variations ranging from 0.2-to 15.0 psu, grows densely in the nutrient enriched zones of the Northern Sector [35]. The Southern Sector of Chilika lagoon supports dense seagrass meadows due to stable salinity regime (mean range ~ 7.0–15.0 psu) with ample light penetration. Both monospecific and mixed seagrass patches (mostly subtidal) with vast and interconnected meadows are reported towards the eastern side of the southern sector [36]. The average and maximum seagrass patch size for the entire lagoon was recorded to be 0.09 and 47.33 km^2^, with seasonal cover ranging between 85.47 (pre-monsoon) and 65.12 km^2^ (post-monsoon). Five dominant species of seagrass (i.e., *Halodule uninervis*, *Halodule pinifolia*, *Halophila ovalis*, *Halophila ovata*, and *Halophila beccarii*) have been reported from this region [37, 38]. The Phailin cyclone, which struck the Chilika coast in October, 2013, significantly reduced the total seagrass cover of the lagoon compared to 2009 [39]. However, a natural recovery of seagrass with an aerial extent of up to 104 km^2^ was reported at Chilika in 2014 [38]. Chilika lagoon has been consistently subjected to natural hazards (viz. cyclone) and anthropogenic pressures (e.g. dredging, brackish water aquaculture, and over-exploitation of resources, etc.). Major anthropogenic activities in the four different sectors of Chilika Lagoon include the following:

1. NS–Runoff from agriculture fields, domestic wastes, aquaculture, dredging, fishing;
2. CS—tourism, fishing, droppings of Guano birds from the bird sanctuary, untreated sewage, effluents from agro-based industries and shrimp processing units;
3. SS- aquaculture, domestic wastes, tourism, fishing; and
4. OC- Tourism, Dredging of the bar mouth [40–42].

### Field sampling

Sampling locations were selected based on the hydrological and environmental/ecological conditions reported in the previous studies in Chilika lagoon [34 and references therein]. Prior to our study, a survey was conducted in the entire lagoon to finalize these locations with special emphasis on the benthic features such as seagrass, submerged aquatic macrophytes etc. (For field surveys and sampling, no specific permissions were required for these locations/activities, as we are an institution under the Ministry of Environment, Forest and Climate Change, Government of India, and are authorized to conduct sampling in this area. We also confirm that the field studies did not involve any endangered or protected species.) Following this, a grid based sampling was undertaken covering the entire lagoon during the dry (May 2014) and wet (September 2014) seasons. Sampling of the entire lagoon covering ~60 x 20 km was done for 5 consecutive days between 9 am to 2 pm during each sampling season, assuming steady state conditions during the sampling period. A total of thirty-five evenly distributed locations were selected and fixed using Global Positioning System (GPS) (Trimble GOXR) (Fig 1 and S1 Table). Considering the spatial heterogeneity, hydrodynamics and aquatic vegetation, sampling locations were grouped into four ecological sectors: i) seagrass dominated Southern Sector (SS; Stations: 1–10, 12 and 17), ii) the Central Sector mostly un-vegetated (CS: Station 11, 13–16, 18–20) represented by the lagoon waters with limited presence of algae and SAV at its periphery; iii) macrophyte-dominated Northern Sector (NS; Stations: 21–31), and iv) tidally active Outer Channel, near the lagoon mouth, (OC; Stations: 32–35) that connects to the Bay of Bengal. Patra et al [43] recently described the presence of four fairly distinctive groups of sampling stations, identical to the present study depending on hydrographic and biogeochemical conditions.

Water samples were collected from the major rivers (n = 13) draining into Chilika Lagoon. Of these, eight of the thirteen rivers (R2 to R9) drain 21.59 x 10^6^ m^3^ d^-1^into the NS with exceptionally high amount of freshwater input, particularly during the wet season. Three rivers (R10 to R12) flow directly into the SS with relatively lesser freshwater inflow (daily mean 1.04 x 10^6^ m^3^ d^-1^), followed by CS (daily mean 0.48 x 10^6^ m^3^ d^-1^) [44].

Surface water samples were collected using 5-liter Niskin water sampler and transferred through silicone tubing into pre-washed High Density Polyethylene bottles (HDPE) bottles. Samples were filtered through combusted 0.45 μm Whatman glass fibre filters (GF/F) and the filtrate was stored in dry ice prior to transport to the laboratory. Water samples were collected in triplicate for dissolved CH_4_ and CO_2_ analysis in gas tight amber colour glass bottles (100 ml) and fixed with 20 μL of saturated HgCl_2_ to arrest microbial activity [45]. Gas samples for ambient measurement were taken using a 20 ml gas-tight syringe and transferred into 12 ml vacuum Exetainers (Labco Limited, USA) for storage. Surface sediment samples were collected using Van-Veen Grab (0.042 m^2^) and scooped using a Teflon coated spatula into airtight polyethylene bags. Water and GHG samples in the SS were mostly collected from mixed meadows of *Halodule* sp. *and Halophila* sp. In this sector, seagrass cover (as %) was estimated at each sampling location during both the seasons, following visual assessment technique of [46]. Two free-divers visually recorded the information of foliage cover density by placing a 0.5 m x 0.5 m quadrat on the lagoon bed. A total of 3 quadrats at each sampling locations were examined in an area approximately 5 m radius. In visual assessment, the observer (diver) ranked the seagrass cover in the quadrat; based on the predetermined reference quadrats with percentage cover ranging from 0% to 100% (very sparse; 0–20%, sparse; 20–40%, medium; 40–60%, dense; 60–80% and very dense; 80–100%).

### Analytical methodology

Meteorological parameters such as wind speed and air temperature were measured *in-situ* using a handheld weather tracker (Kestrel 4500 NV) and were mostly used to calculate various air-water GHG exchange fluxes. *In-situ* measurements of water quality (salinity, dissolved oxygen, chlorophyll-a (Chl-a) and temperature) were carried out using a pre-calibrated water quality probe (HYDROLAB). pH was measured immediately after collection using glass combination electrode (DGi115-SC) and a pH meter (Metler Toledo G20) calibrated through NBS scale (US National Bureau of Standards), with an accuracy and reproducibility of ± 0.005 pH units. Nutrients (nitrate, nitrite, phosphate, silicate and ammonium) were analysed using an automated continuous flow nutrient analyzer (Skalar San++) system calibrated using appropriate standards. Total alkalinity (TA) was determined through potentiometric titration [47] using Metler Toledo Compact Titrator G20. Acidified water samples (with 10% orthophosphoric acid to maintain the pH between 2 and 3) were used for measurement of Dissolved Organic carbon (DOC) using a Total Organic Carbon (TOC) Analyzer (Elementar Vario EL III) following high temperature catalytic oxidation method. Station 4 from SS and 30 from NS were the two representative locations studied for seagrass community metabolism. Net Community Production (NCP), which is the difference between gross primary productivity (GPP) and community respiration (CR), was calculated based on the open water O_2_ mass balance technique (24 hours of *in-situ* observation) [48].

The HYDROLAB Sonde with an optical probe was used to measure DO at 0.4 m and 0.1 m above the seagrass beds at 5-minute intervals, and then we integrated the data over a 24-hour period. The data sets were combined to provide the vertically-integrated gradient of O_2_ (expressed in mmol m^-2^) on an hourly basis. Oxygen content of the whole water column (~2 m) was used for the computation of productivity, as both sampling locations were in general vertically isothermal. Computations were made considering a 24-h cycle starting at sunrise of any given day.

pCO_2_ was calculated indirectly from pH and TA using the CO_2_SYS program [49]. Excess carbon dioxide (ECO_2_ in μmol kg^–1^), defined as the quantity of Dissolved Inorganic Carbon (DIC) that must be released as CO_2_ to the atmosphere to achieve complete air–water equilibrium, was also calculated using CO_2_SYS program. Dissolved methane was determined by the headspace equilibration technique described by [50]. A predetermined volume (50 ml) was equilibrated with an equal volume of helium in a gas-tight syringe using a wrist action shaker (KEMI) at room temperature for 10 minutes at 100 RPM.

The equilibrated headspace gas samples were analysed for methane using a gas chromatograph (Shimadzu 2010 Plus) fitted with a stainless steel Poropak QS packed column at 60°C and flame ionization detector (FID) temperature maintained at 250°C. The precision of repeated analysis of gas samples was well within 5%. Reference gas standards of 2.5 ppmv and 10 ppmv (Scott Specialty Gases) were analysed after every 5 samples. Air-water fluxes were calculated as FTG = *k* ΔTG [51, 52] where FTG is the flux of CO_2_ and CH_4_ expressed in mmol C m^-2^ d^-1^, k is the gas transfer velocity [51] normalized to the Schmidt number of 600 in m d^-1^ [53], and ΔTG is the difference in the surface water GHG concentration to atmospheric air GHG concentration at equilibrium. The gas transfer velocity (k) was calculated using wind speed at 10 m (u_10_, in m s^-1^), water current (w in cm s^-1^) and water column depth (h) following the equation by [51]:

$$k_{600}=0.24+04126\;w^{0.5}h^{-0.5}+0.610u_{10}$$

Positive values of FTG denote net gas exchange of water to the atmosphere and vice versa. Mean CO_2_ and CH_4_ emissions from each zone were converted into CO_2_ equivalents (CO_2_e) based on the 100-year Global Warming Potential (GWP) factors listed [17] (28 for CH_4_). Sediment organic carbon (SOC) was determined using CHNS Elemental Analyzer (Thermo Flash 2000) with an accuracy of ±0.1–0.5% as assessed by replicate analyses of certified reference material (Soil Reference Material NCS, Thermo).

ArcGIS version 10.5.1 was used to represent the sampling locations in the study area. Analysis of variance (ANOVA) was used to determine significant differences in the physico-chemical and GHG concentrations (CO_2_ and CH_4_) among the sectors. Prior to the ANOVA tests, the water quality data were tested using the Kolmogorov–Smirnoff test to confirm normal distribution, and Levene’s test (p>0.05) to assess the homogeneity of variances without any transformation. Correlation analysis was performed to identify inter-parameter relationship between the physico-chemical parameters and the GHGs. Linear or nonlinear regression models were used in order to explain the spatio-temporal variability in GHGs.

## Results

### Lagoon environment

Physical: The meteorological parameters showed considerable variations largely due the seasonal change of the inter-tropical convergence zone. Higher air temperature was recorded during dry period (30.56 ± 0.78°C) compared to the wet period (28.28 ± 0.54°C). Both air and water temperature showed a similar seasonal pattern with no significant spatial variations (p>0.05). The physico-chemical parameters and nutrient concentrations of surface water in the different ecological zones of Chilika Lagoon varied widely from the dry to wet seasons (Table 1 and S2 Table). Wind speed, however, remained more or less constant throughout the study period (1.94 to 4.06 m s^-1^), with the maximum annual mean wind speed observed at the Outer Channel (Table 1). Higher gas transfer velocity (11.0 cm hr^-1^) was recorded during wet season compared to the dry season (5.5 cm hr^-1^) in the lagoon. The mean k_600_ [51] value for Chilika Lagoon was estimated to be 7.4 ± 2.8 cm hr^-1^.

**Table 1:** Mean (± SD) seasonal variation of physico-chemical parameters, nutrients and sediment organic carbon of surface waters of the Chilika Lagoon; numbers in parentheses indicate the observed range.

	DRY SEASON				WET SEASON			
Parameters	Southern Sector (SS) n = 12	Central Sector (CS) n = 8	Northern Sector (NS) n = 11	Outer Channel (OC) n = 4	Southern Sector (SS) n = 12	Central Sector (CS) n = 8	Northern Sector (NS) n = 11	Outer Channel (OC) n = 4
**Water Temp.** **(°C)**	30.0 ± 0.68 (28.9–31.2)	29.9 ± 0.80 (29.1–31.2)	30.7 ± 0.32 (30.2–31.4)	30.2 ± 0.72 (29.7–31.3)	27.94± 0.70 (26.3–28.8)	27.4± 0.69 (25.9–28.0)	26.5± 0.84 (25.5–28.0)	27.7± 0.20 (27.5–27.9)
**Wind Speed** **(m s**^**-1**^**)**	3.93 ± 2.3 (1.30–7.40)	2.29 ± 1.2 (0.500–4.00)	2.22 ± 0.92 (1.30–4.10)	4.06 ± 0.23 (3.76–4.24)	1.94 ± 0.65 (1.20–3.00)	3.43 ± 1.5 (0.560–5.31)	3.94 ± 0.96 (2.70–5.68)	3.88 ± 1.6 (2.10–5.20)
**Salinity** **(PSU)**	15.1 ± 2.9 (11.2–20.6)	23.4 ± 6.0 (17.6–32.4)	17.4 ± 5.1 (7.5–25.9)	33.7 ± 0.26 (33.4–33.9)	6.65 ± 1.0 (4.96–7.97)	3.52 ± 1.9 (0.550–5.65)	1.74 ± 1.7 (0.130–5.22)	10.0 ± 4.4 (6.99–16.5)
**pH**	8.29 ± 0.21 (8.02–8.68)	7.98 ± 0.07 (7.87–8.10)	7.95 ± 0.12 (7.65–8.10)	7.99 ± 0.06 (7.94–8.07)	8.01 ± 0.07 (7.93–8.17)	7.75 ± 0.10 (7.56–7.85)	7.39 ± 0.07 (7.28–7.49)	7.84 ± 0.14 (7.70–7.99)
**Dissolved Oxygen** **(% Sat.)**	115 ± 12 (95.9–133)	111 ± 12 (90.6–124)	109 ± 37 (69.5–194)	96.3 ± 16 (84.0–120)	102 ± 8.8 (93.6–126)	95.0 ± 5.4 (89.1–101)	93.6 ± 4.5 (87.6–102)	94.4 ± 4.4 (89.4–100)
**DIN** **(μM L**^**-1**^**)**	4.36 ± 1.7 (1.40–7.53)	4.61 ± 1.4 (3.11–7.12)	6.88 ± 1.2 (5.01–9.19)	4.89 ± 1.2 (3.79–6.13)	6.73 ± 0.63 (5.84–7.75)	7.31 ± 1.1 (6.24–9.74)	8.70 ± 1.7 (5.71–11.6)	6.43 ± 1.0 (5.36–7.64)
**DIP** **(μM L**^**-1**^**)**	0.701 ± 0.13 (0.491–0.865)	0.888 ± 0.35 (0.259–1.18)	0.813 ± 0.63 (0.385–2.14)	0.389 ± 0.05 (0.333–0.444)	0.525 ± 0.13 (0.232–0.679)	0.820 ± 1.1 (0.132–3.38)	0.898 ± 0.34 (0.539–1.54)	0.682 ± 0.53 (0.139–1.20)
**DSi** **(μM L**^**-1**^**)**	53.1 ± 6.5 (40.6–64.6)	59.3 ± 29 (14.0–89.9)	71.7 ± 32 (35.7–138)	23.6 ± 5.9 (17.1–31.0)	84.0 ± 22 (47.4–133)	127 ± 28 (73.4–159)	138 ± 28 (82.9–171)	112 ± 36 (74.7–161)
**SPM** **(mg L**^**-1**^**)**	53.6 ± 32 (26.7–115)	79.0 ± 43 (29.5–158)	90.3 ± 54 (27.1–168)	68.2 ± 27 (27.7–83.9)	84.0 ± 35 (44.0–144)	155.0 ± 58 (105–280)	211 ± 112 (41.4–351)	98.8 ± 13 (84.0–113)
**Chlorophyll-a** **(mg m**^**-3**^**)**	8.65 ± 3.7 (1.18–12.5)	6.24 ± 1.4 (4.43–8.69)	7.23 ± 1.4 (4.97–8.80)	2.43 ± 0.62 (1.66–3.18)	4.14 ± 1.7 (1.89–6.87)	3.28 ± 1.1 (2.11–5.32)	5.81 ± 1.1 (4.24–8.10)	1.63 ± 0.57 (0.980–2.23)
**DOC** **(mg L**^**-1**^**)**	2.56 ± 0.47 (1.93–3.78)	1.87 ± 0.40 (1.23–2.34)	2.77 ± 0.45 (1.95–3.41)	0.979 ± 0.10 (0.839–1.05)	3.12 ± 0.83 (1.81–4.45)	2.89 ± 0.27 (2.30–3.10)	4.16 ± 0.58 (3.26–5.12)	1.41 ± 0.21 (1.20–1.66)
**DIC** **(μM/ kg)**	2226 ± 150 (1889–2553)	1878 ± 110 (1730–2008)	1923 ± 340 (1389–2301)	2095 ± 59 (2026–2147)	1930 ± 69 (1809–2071)	1731 ± 370 (1186–2136)	1905 ± 170 (1704–2194)	1751 ± 100 (1668–1888)
**SOC (%)**	0.893 ± 0.33 (0.552–1.46)	0.630 ± 0.38 (0.086–1.10)	0.833 ± 0.48 (0.100–1.52)	0.407 ± 0.15 (0.235–0.547)	0.976 ± 0.21 (0.772–1.35)	0.940 ± 0.22 (0.526–1.14)	1.41 ± 0.41 (0.313–1.79)	0.601 ± 0.30 (0.292–0.892)

DIN = Dissolved Inorganic Nitrogen; DIP = Dissolved Inorganic Phosphate; DSi = Dissolved Inorganic Silicate; SPM = Suspended Particulate Matter; DOC = Dissolved Organic Carbon; DIC = Dissolved Inorganic Carbon; SOC = Sediment Organic Carbon

Hydrological: A majority (95%) of the inflow from the rivers occurred during the wet season (166.8 x 10^6^ m^3^ d^-1^) with relatively meagre inputs during the dry season (3.1 x 10^6^ m^3^ d^-1^; [33]). Salinity of the rivers was always <0.1 PSU, during both seasons. In the lagoon, however, there were significant spatio-temporal variations (p<0.05) in salinity that can be attributed primarily to localized differences in river discharge to the lagoon.

Dissolved Oxygen: Mean dissolved oxygen saturation of the lagoon surface waters varied from marginal under-saturation (96%) to super-saturation (115%) during the dry season, and was predominantly under-saturated (93% to 102%) during the wet season (Table 1). Exceptionally high O_2_ saturation (194%), recorded at station 25 in the northern sector during dry season could be attributed to direct influence of the biological production from the benthic macrophytes. Among the sectors, the highest mean dissolved oxygen saturation was observed consistently at the SS. However, this spatial variation in DO saturation was statistically insignificant (p = 0.093). Further, significant inter-seasonal variations in DO saturation were recorded for SS (p = 0.009) and CS (p = 0.004).

Suspended Sediments: Suspended sediment concentrations (SPM) varied spatially with highest SPM in the northern sector and follows the order: NS>CS>OC>SS (Table 1). During wet season, suspended sediments were at least three times higher in the NS than SS, presumably due to the large influx from the rivers. This caused a decrease in photic depth and lowered productivity in the NS. This phenomenon was not distinct in the dry season as the SPM values among the sectors varied insignificantly.

Nutrient and primary producers: Using the atomic Si:N:P ratio of 16:16:1 [54] as a criterion for balanced nutrient composition, one can distinguish between silicon, nitrogen or phosphorous deficient system. The entire Chilika lagoon was nitrogen limited throughout the year (84:6:1), and the conditions were more severe during the dry season (52:5:1), when the mean lagoon salinity was relatively higher. Among sectors, N:P ratio was the highest at OC during dry season in the following sequence: OC (12.5:1) >NS (8.4:1) >SS (6.2:1) >CS (5.2:1). During the wet season, a two fold increase in N:P ratio at SS was observed and follows the order SS (12.8:1) >NS (9.7:1) >OC (9.4:1) >CS (8.9:1). N:P ratio at CS remained consistently low during both seasons.

Average Chl-a concentration showed an increasing trend from wet to dry season throughout the lagoon. The highest mean Chl-a concentration (8.65 ± 3.7 mg m^-3^) was found in SS, where the influence of freshwater and suspended particles was relatively low during the dry season (Table 1). The spatio-temporal pattern of dissolved organic carbon (DOC) revealed that the highest concentrations were from the NS (4.16 ± 0.58 mg l^-1^) during the high flow period (wet).

Net Community Production: O_2_ concentrations, used for the measurement of NCP at the diurnal stations, showed a similar temporal pattern, which indicated strong biological activities at both the stations. At SS, the highest increase in hourly O_2_ concentrations was recorded between 8 am and 9 am during both dry (51.3mM m^-2^ hr^-1^) and wet (46.16 mM m^-2^ hr^-1^) season. This highest increase in hourly O_2_ concentrations during daytime for NS was relatively low during both dry (11.43 mM m^-2^ hr^-1^) and wet (12.65 mM m^-2^ hr^-1^) seasons. The net community production (NCP), which regulates pCO_2_ concentrations through biological consumption/release, was positive at the SS, with higher levels during the dry season (70.7 mmol C m^-2^ d^-1^) compared to the wet season (52.6 mmol C m^-2^ d^-1^). On the contrary, NCP values were distinctly lower in the NS with -13.26 and 2.36 mmol C m^-2^ d^-1^, during the wet and dry seasons, respectively.

### Trace gas dynamics in the Lagoon

#### CO_2_

**Fig 2:**
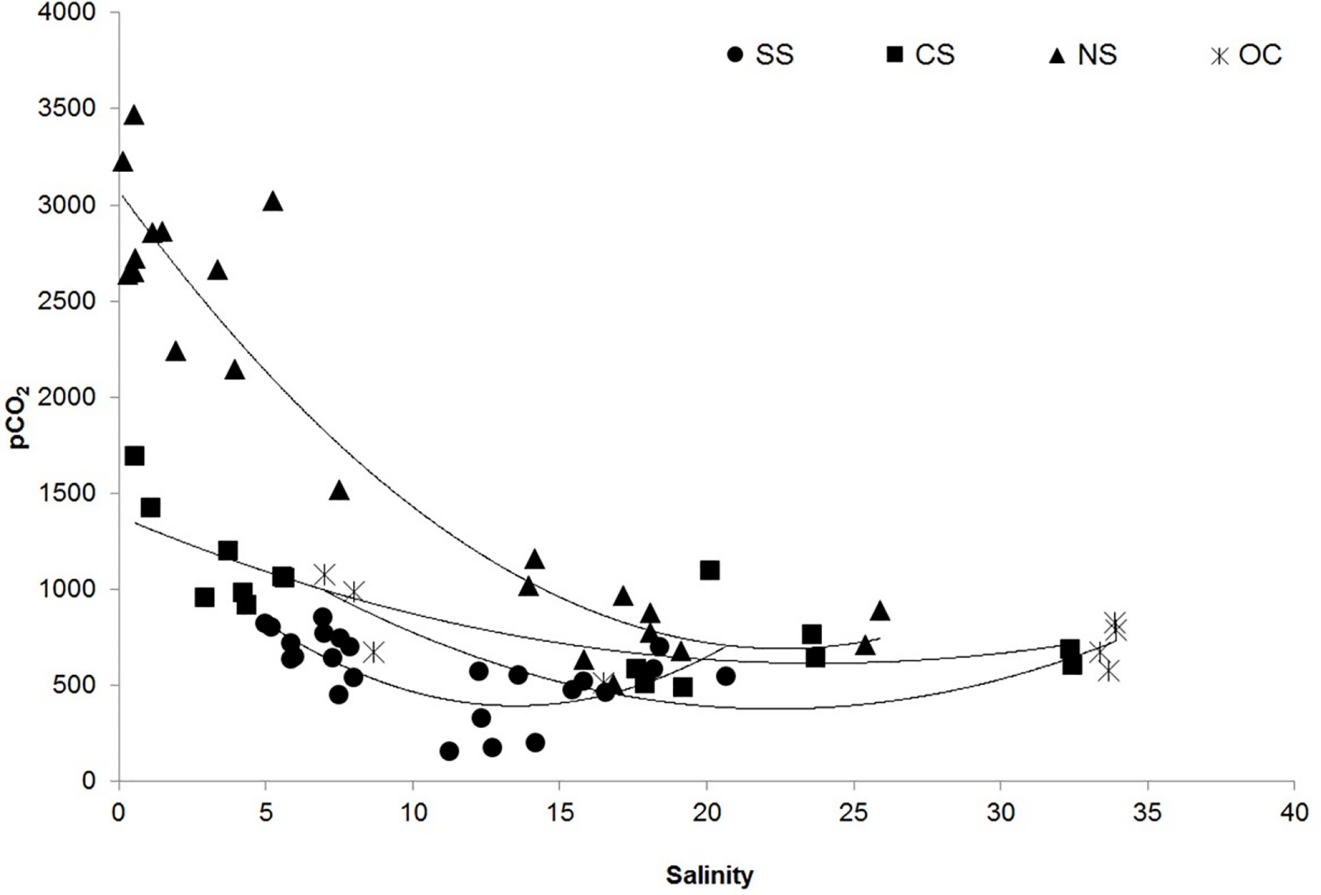
Distribution of *p*CO_2_ with respect to salinity in different sectors of Chilika Lagoon; SS = Southern Sector; CS = Central Sector; NS = Northern Sector and OC = Outer Channel.

Variations in dissolved CO_2_ (pCO_2_) and CH_4_ concentrations and their fluxes for the different ecological sectors of Chilika are provided in Table 2 (S3 Table). The annual range of pCO_2_ varied between 159 and 858 μatm at the SS, and between 512 and 1,100 μatm at the OC. The annual range of pCO_2_ was considerably higher for CS (487 to 1700 μatm) and NS (508 to 3,470 μatm). Rivers draining into the lagoon were extremely supersaturated (i.e., 593 to 4,370%) with respect to atmospheric CO_2_, with pCO_2_ ranging between 2,900 and 16,600 μatm (Table 3 and S3 Table). A strong positive correlation was observed between river discharge and pCO_2_ levels, which was most pronounced in the NS. The second-order polynomial equations with the highest coefficient of determination (R^2^) were chosen as best fit to explain the relationship between salinity and pCO_2_ at all the sectors (Fig 2 and S2 Table; Table 4 and S2 Table).

**Table 2:** Mean (± SD) seasonal variation of CO_2_ and CH_4_ concentrations and fluxes from surface waters of the Chilika Lagoon; numbers in parentheses indicate the range observed.

DRY SEASON					WET SEASON			
GHG	Southern sector (SS) n = 12	Central sector (CS) n = 8	Northern sector (NS) n = 11	Outer channel (OC) n = 4	Southern sector (SS) n = 12	Central sector (CS) n = 8	Northern sector (NS) n = 11	Outer channel (OC) n = 4
***p*CO**_**2**_ **(**μ**atm)**	442 ± 180 (159–703)	673 ± 190 (487–1100)	886 ± 280 (508–1520)	716 ± 110 (576–826)	697± 120 (456–858)	1161± 270 (920–1700)	2773 ± 390 (2145–3470)	813 ± 260 (512–1100)
***CO***_***2***_ ***Flux*** **(mmol m**^**-2**^ **d**^**-1**^**)**	1.19 ± 16 (-33.9–21.1)	19.4 ± 22 (2.59–72.6)	29.0 ± 16 (4.94–66.7)	28.9 ± 9.4 (17.8–36.7)	18.4 ± 8.8 (3.11–29.8)	77.4 ± 52 (17.1–186)	264 ± 74 (188–376)	37.0 ± 18 (14.5–59.6)
**CH**_**4**_ **(nM L**^**-1**^**)**	33.2 ± 25 (10.5–80.2)	22.5 ± 7.4 (15.1–34.0)	29.8 ± 20 (7.32–77.6)	18.7 ± 4.0 (14.4–23.6)	43.6 ± 31 (16.0–127)	16.0 ± 8.5 (9.05–35.4)	33.4 ± 16 (10.1–53.5)	21.4 ± 10.1 (14.7–36.4)
**CH**_**4**_ **Flux** **(mmol m**^**-2**^ **d**^**-1**^**)**	0.135 ± 0.13 (0.030–0.391)	0.056 ± 0.03 (0.014–0.103)	0.078 ± 0.05 (0.013–0.199)	0.064 ± 0.02 (0.047–0.089)	0.100 ± 0.07 (0.030–0.257)	0.052 ± 0.04 (0.011–0.141)	0.132 ± 0.08 (0.024–0.258)	0.075 ± 0.01 (0.056–0.090)

**Table 3:** Mean (± SD) seasonal variation of CO_2_ and CH_4_ concentrations and fluxes from surface waters of rivers entering the Chilika Lagoon; numbers in parentheses indicate the range observed.

	DRY SEASON			WET SEASON		
GHG	Southern Sector (SS) n = 3	Central Sector (CS) n = 2	Northern Sector (NS) n = 8	Southern Sector (SS) n = 3	Central Sector (CS) n = 2	Northern Sector (NS) n = 8
***p*CO**_**2**_ **(**μ**atm)**	6460 ± 2400 (4360–9150)	6190 ± 4800 (2800–9580)	6200 ± 2100 (2900–9100)	14700 ± 2700 (12700–16600)	8260 ± 320 (8040–8440)	10900 ± 3100 (4580–13900)
***CO***_***2***_ ***Flux*** **(mmol m**^**-2**^ **d**^**-1**^**)**	540.5 ± 337.6 (293.8–925.3)	343 ± 290 (141–546)	427 ± 170 (151–648)	617 ± 48 (583–651)	482 ± 33 (459–505)	1180 ± 750 (454–2780)
**CH**_**4**_ **(nM L**^**-1**^**)**	192 ± 240 (22.2–471)	98.2 ± 14 (88.1–108)	302 ± 170 (39.4–581)	252 ± 59 (205–319)	176 ± 7.3 (171–181)	157 ± 95 (13.4–337)
**CH**_**4**_ **Flux** **(mmol m**^**-2**^ **d**^**-1**^**)**	0.563 ± 0.60 (0.105–1.24)	0.265 ± 0.06 (0.224–0.306)	1.00 ± 0.54 (0.190–1.81)	0.660 ± 0.12 (0.560–0.798)	0.334 ± 0.01 (0.331–0.338)	0.394 ± 0.19 (0.031–0.674)

**Table 4:** Correlation results between pCO_2_ and salinity of all the sectors of the Chilika Lagoon.

Sector	n	r^2^	Polynomial fit
SS	16	0.54	y = 6.026x^2^–162.5x + 1491
CS	12	0.67	y = 1.349x^2^–64.24x + 1380
NS	22	0.91	y = 4.673x^2^–210.9x + 3073
OC	8	0.69	y = 2.621x^2^–117.0x + 1684.

**Fig 3:**
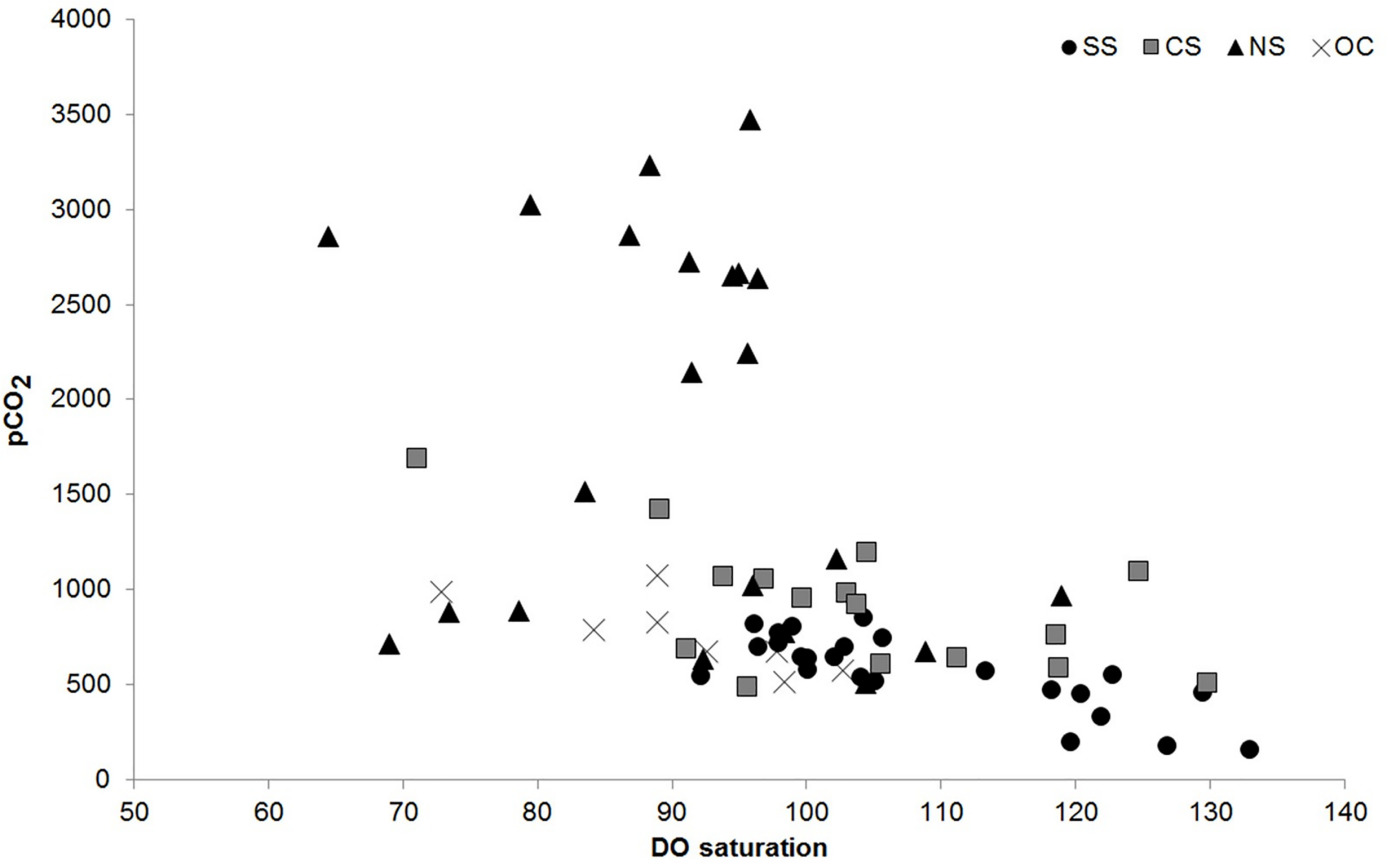
Distribution of *p*CO_2_ with respect to DO saturation at different parts of the Chilika Lagoon; SS = Southern Sector; CS = Central Sector; NS = Northern Sector and OC = Outer Channel.

**Fig 4:**
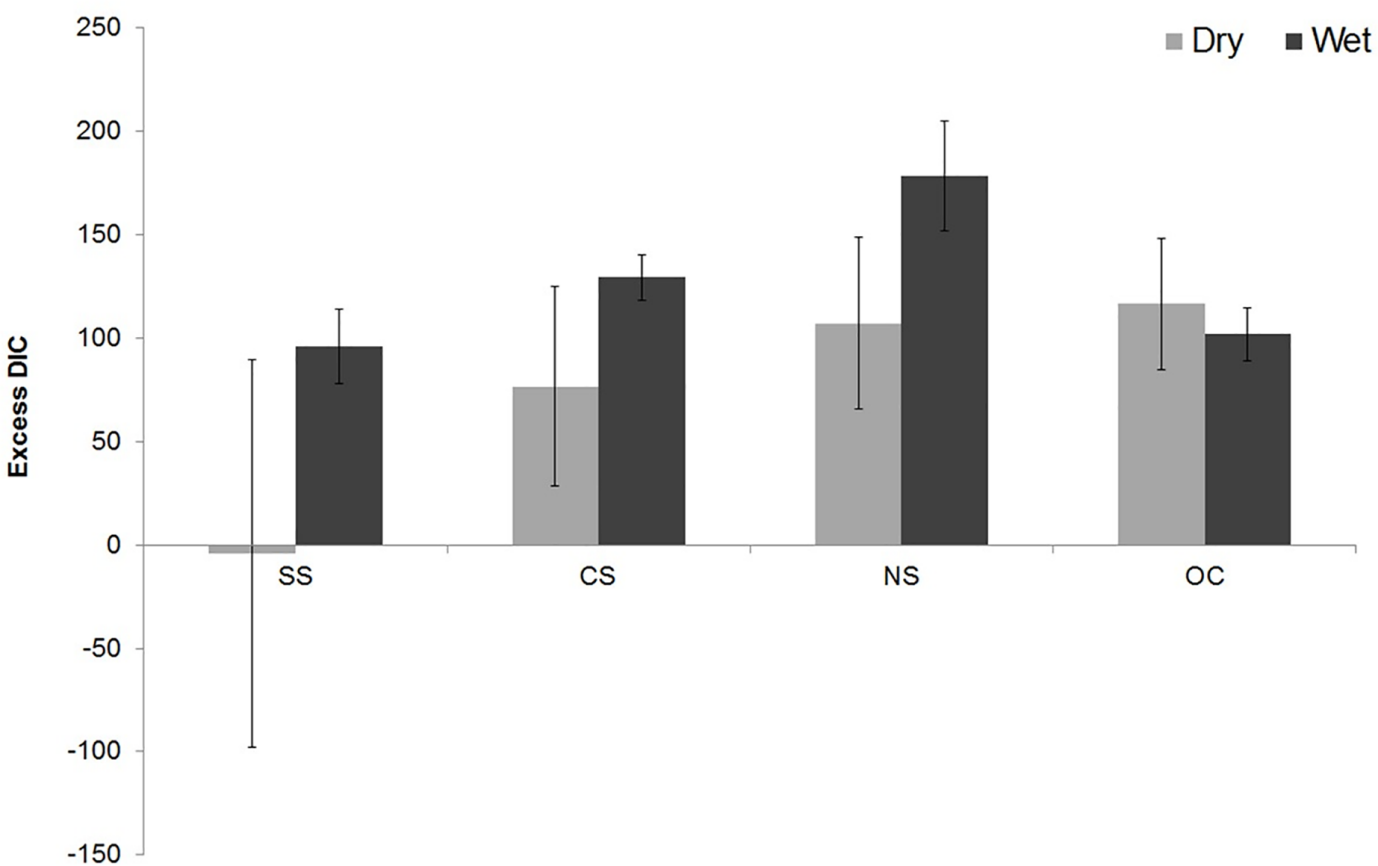
Excess DIC distribution during the dry and wet seasons at different sectors of the Chilika Lagoon; SS = Southern Sector; CS = Central Sector; NS = Northern Sector and OC = Outer Channel. (Error bars represent Standard Deviation).

A negative correlation was observed between pCO_2_ and DO saturation (Fig 3 and S2 Table), which indicated that high saturation of DO was associated with low concentrations of pCO_2_, which was most significant at the SS (R^2^ = 0.63, p <0.001, F = 39.3), and follows the order OC (R^2^ = 0.58, p = 0.029, F = 8.16) and CS (R^2^ = 0.36, p = 0.015, F = 7.96) and the relationship was insignificant at NS (R^2^ = 0.09, p = 0.18, F = 2.17). Excess DIC, which is derived as a result of biological processes, was lowest at the SS (average -4 ± 51.6 μM kg^-1^) during the dry season. An overall increase in excess DIC was recorded during wet season with the highest and lowest values from NS (178 ± 27.3 μM kg^-1^) and SS (98.2 ± 21.5 μM kg^-1^) (Fig 4 and S2 Table). Significant positive correlations between DOC and pCO_2_ were recorded for all the sectors, (SS, r = 0.58, CS, r = 0.69; NS, r = 0.85; and OC, r = 0.66) with variability in shape of the curves. During the dry season, highest CO_2_ fluxes (FCO_2_) were observed at the NS, followed by OC>CS>SS. In the wet season, FCO_2_ followed the order NS>CS>OC>SS (Tables 2 and 3). During both seasons, FCO_2_ was lowest at SS. We observed largest flux of CO_2_ at NS during the high flow (wet) period, and was estimated to be at least 9 times higher than the low flow (dry) period.

#### CH_4_

The Chilika lagoon waters were always super-saturated with dissolved CH_4_ with respect to atmospheric equilibrium (Table 2). Highest dissolved CH_4_ concentrations were observed in the seagrass dominated SS with marginal temporal variation between the dry (33.2 nM L^-1^) and wet (43.6 nM L^-1^) seasons. CH_4_ concentration in river surface waters (Tables 2 and 3) contributing to the lagoon (mean concentration of all rivers: 197 nM L^-1^) was over seven folds higher than the lagoon water concentration (mean of all sectors 27 nM L^-1^). During the dry season, the highest CH_4_ concentration was recorded in the rivers draining to the NS followed by the rivers draining into SS and CS. In the wet season, rivers draining into the SS had the highest concentration of CH_4_, which influenced the seagrass meadows directly. Varying quantities in freshwater inputs to the three sectors of the lagoon could be a significant confounding variable that can limit the understanding of CH_4_ flux associated with seagrass meadows [55].

**Fig 5:**
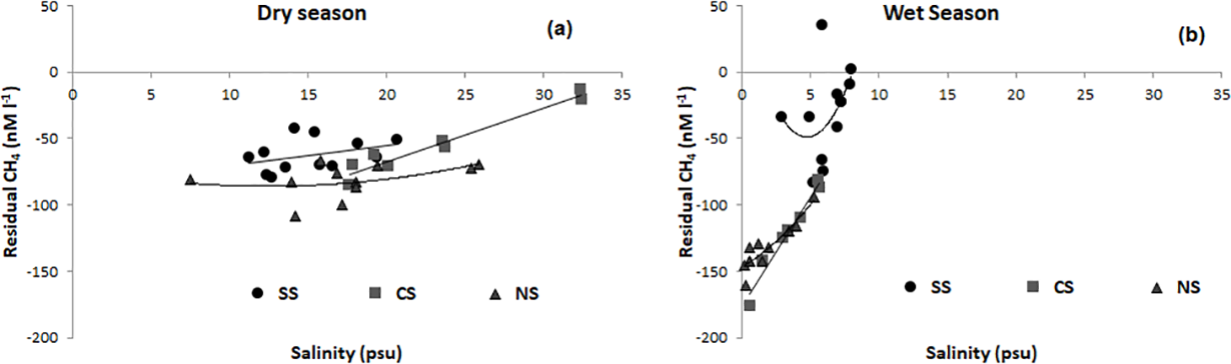
Residual methane between observed concentrations and values estimated from conservative mixing with respect to salinity during a) dry and b) wet seasons in different sectors of the Chilika Lagoon; SS = Southern Sector; CS = Central Sector; NS = Northern Sector.

Further, to understand the role of seagrass in regulating the CH_4_ fluxes in the lagoon, residual CH_4_ concentrations relative to the conservative mixing of seawater and freshwater were plotted against salinity (Fig 5 and S2 Table; Table 5). The observed CH_4_ concentrations and the conservative mixing line indicated the intensity of CH_4_ sink in the lagoon. No significant relationship between salinity and CH_4_ residuals were observed at SS during both the seasons, whereas significant linear relationship (R^2^ = 0.83, F = 43.36, p<0.01) was recorded for NS only during wet season. For CS, this relationship was linear during both dry (R^2^ = 0.94, F = 101.37, p<0.01) and wet season (R^2^ = 0.96, F = 158.1, p<0.01).

**Table 5:** Correlation results between CH_4_ residuals and salinity (S) of all the sectors of the Chilika Lagoon.

Dry season			
Sector	n	r^2^	Equation
SS	8	14.2	CH_4_ residuals = 1.52S - 85.5
CS	7	91	CH_4_ residuals = 4.02S + 17
NS	11	21.6	CH_4_ residuals = 0.09S^2^–2.3S - 71.3
**Wet season**			
SS	8	21.8	CH_4_ residuals = 4.4S2–41.6S + 49.8
CS	7	96.3	CH_4_ residuals = 16.38S - 176.2
NS	11	83.4	CH_4_ residuals = 0.64S^2^ + 6.22S - 146.8

**Fig 6:**
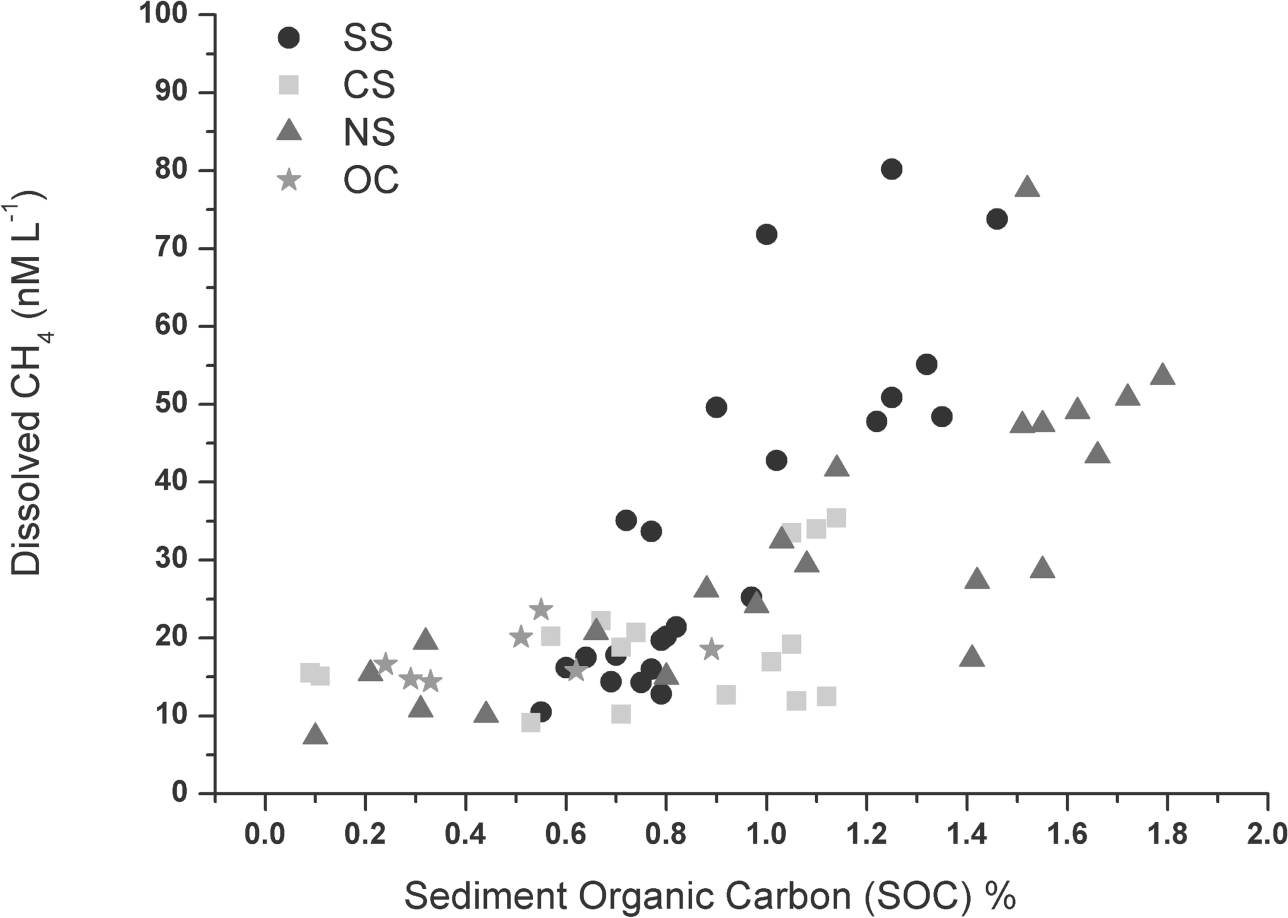
Influence of sediment organic carbon (SOC) on the distribution of dissolved CH_4_ in different sectors of the Chilika Lagoon; SS = Southern Sector; CS = Central Sector; NS = Northern Sector and OC = Outer Channel.

Sediment organic Carbon: In general, sediment organic carbon (SOC) was low and the annual range varied from 0.24 to 0.89% at the OC and from 0.10 to 1.79% at the NS. The lowest SOC was recorded at CS with a range between 0.09 and 1.14%, whereas for SS the annual range varied from 0.55 to 1.46% (Table 1). Further, SOC concentrations were relatively higher in the sectors dominated by macrophyte and seagrass. Lowest concentrations were recorded in the tidally influenced OC, where sand was dominant. Positive correlation was observed between SOC and dissolved CH_4_ concentrations at both SS (R^2^ = 0.61, p<0.001, F = 34.62), NS (R^2^ = 0.62, p<0.001, F = 32.16) and OC (R^2^ = 0.72, p = 0.016, F = 12.60) (Fig 6 and S2 Table). The slope of the relationship was maximum in the NS (0.022) followed by OC (0.017), CS (0.011) and SS (0.007).

**Fig 7:**
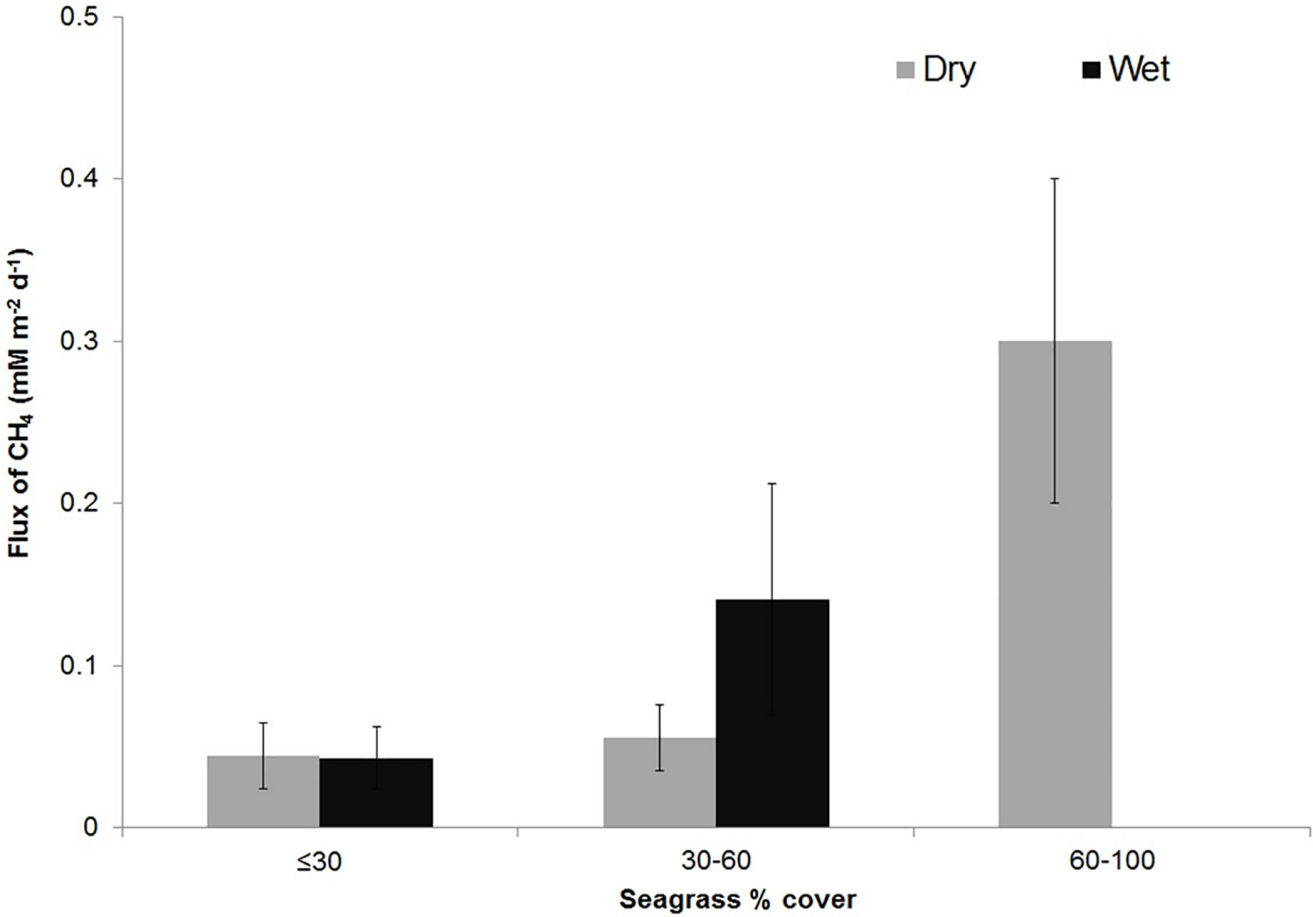
Influence of seagrass cover (%) in Southern sector on CH_4_ flux (mmol m^-2^ d^-1^). During wet season, seagrass cover never surpasses 60%. Seagrass density is reduced drastically due to its decay (influenced by higher freshwater input) contributing to higher CH_4_ emission rates in the wet season (Error bars represent Standard Deviation).

The seagrass covers in the sampling stations of SS ranged between 30–100% with a mean of 55% during dry season, and between 25 and 45% (mean = 37%) during wet season. Natural seasonal cycles of seagrass density generally followed a pattern of high spatial cover during dry season, influenced by high salinity and low spatial cover in the wet season due to death and decay (density <60%). The relationship between seasonal seagrass density and CH_4_ emissions is explained in Fig 7 (S4 Table). Higher CH_4_ fluxes from the sampling stations with denser seagrass cover in the SS indicated the potential role of the meadows in the regulation of CH_4_ fluxes.

**Fig 8:**
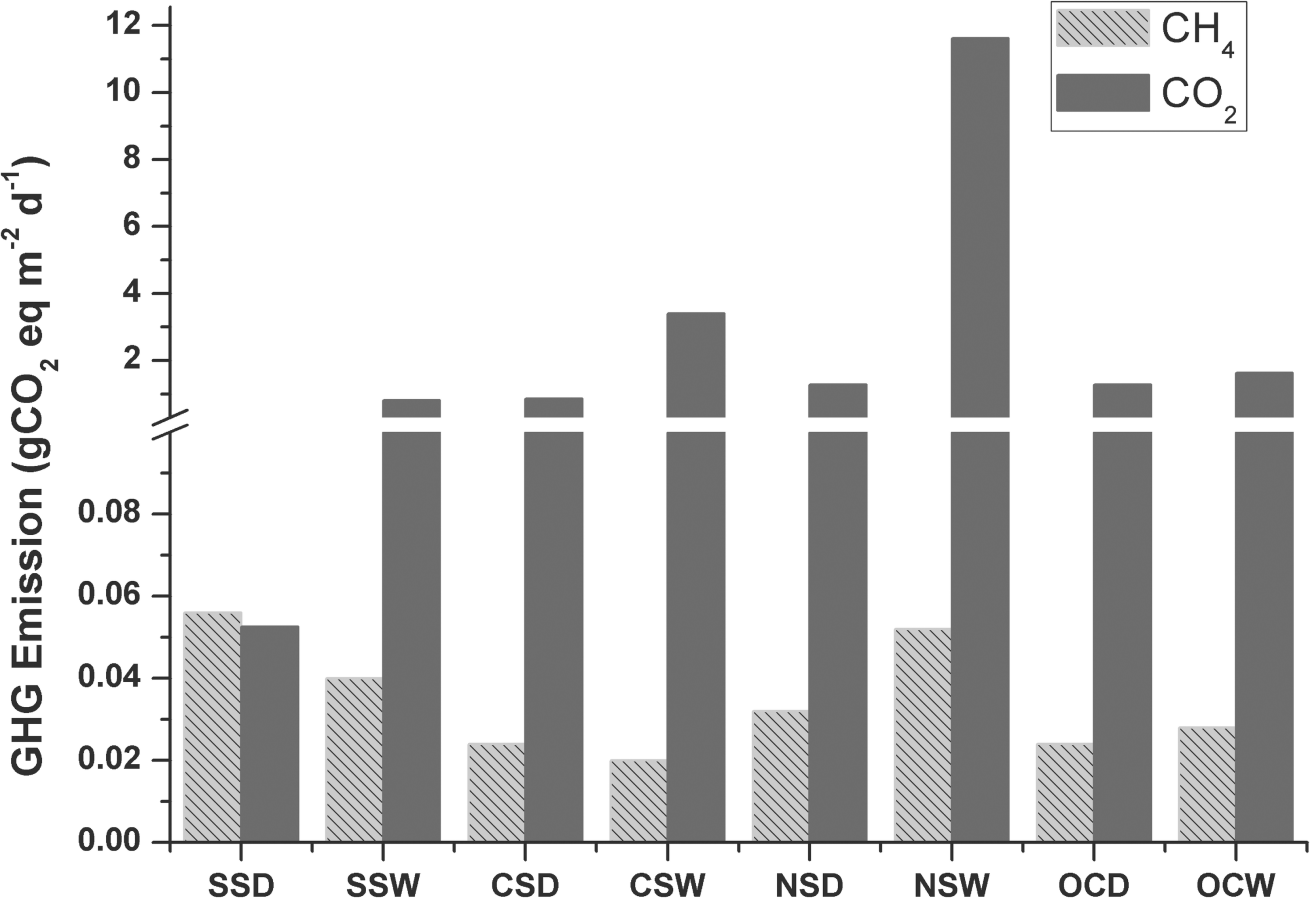
Air- water exchange flux of CO_2_ and CH_4_ in terms of CO_2_ equivalents (CO_2_e) during present study from different parts of the Chilika Lagoon. SSD = Southern Sector Dry; SSW = Southern sector Wet; CSD = Central Sector Dry; CSW = Central Sector Wet; NSD = Northern Sector Dry; NSW = Northern Sector Wet; OCD = Outer Channel Dry and OCW = Outer Channel Wet; SS = Southern Sector, CS = Central Sector; NS = Northern Sector; OC = Outer Channel.

**Fig 9:**
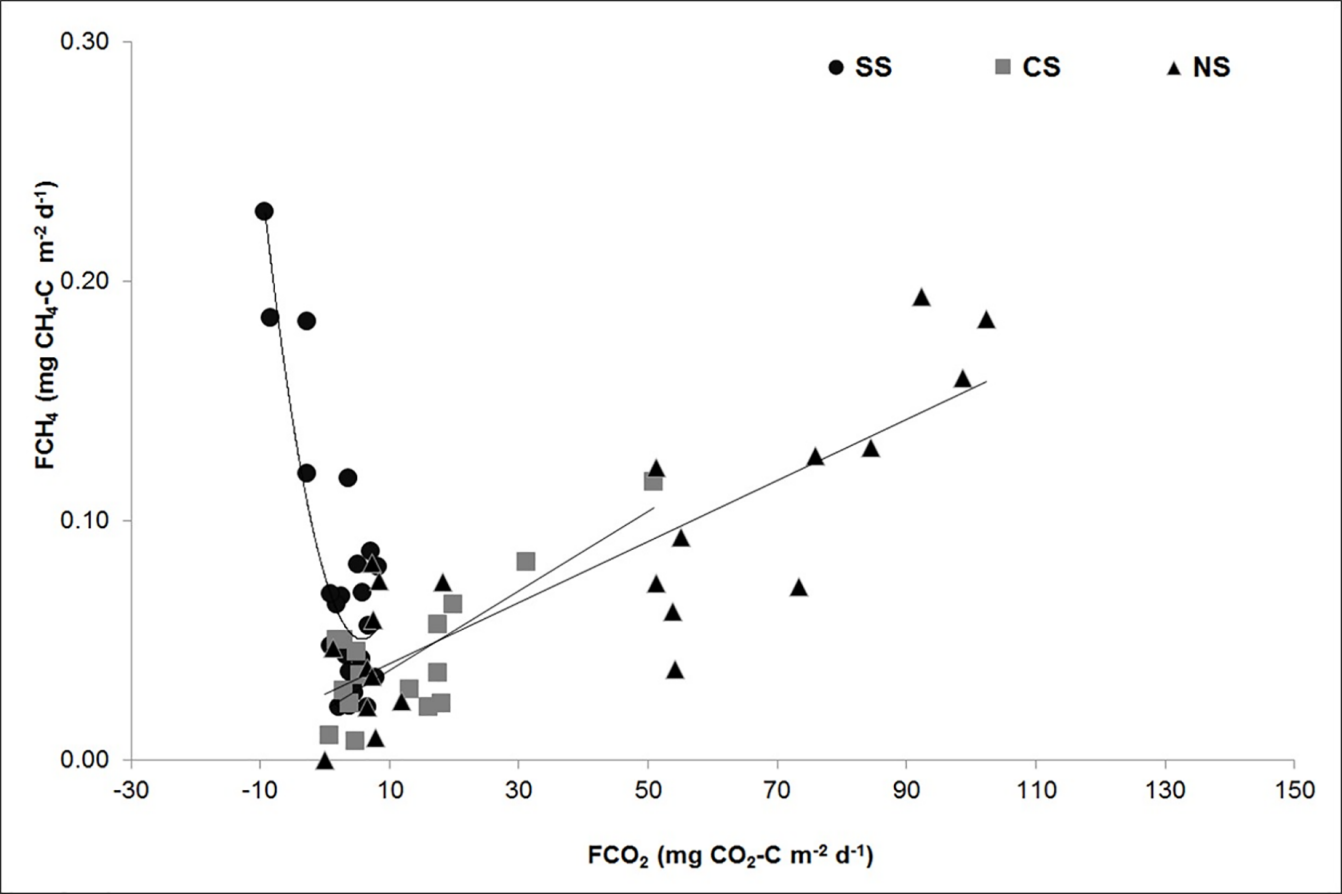
Relationship between CO_2_ and CH_4_ fluxes in Seagrass dominated southern sector, Lagoon waters of central sector and Macrophyte dominated northern sector of the Chilika Lagoon.

Flux values of CO_2_ and CH_4_ were converted into corresponding CO_2_ equivalents (Fig 8). The cumulative GHG flux was highest at NS (11.7 g CO_2_e m^-2^ d^-1^) during the wet season and the least at SS. Air-water fluxes of both CO_2_ and CH_4_ were plotted to determine their inter-relationship among the various sectors (Fig 9 and S3 Table; Table 6). A second-order polynomial curve is used to explain the relation between air-water fluxes of both CO_2_ and CH_4_ in the SS (Fig 9). The results indicated higher CH_4_ fluxes from the areas with high CO_2_ sink, whereas the correlation is weak with increasing CO_2_ fluxes. For the un-vegetated CS waters and macrophyte dominated NS, the relationship between FCO_2_ and FCH_4_ was moderately positive and linear (R^2^ = 0.59, F = 15.19, p = 0.008 and R^2^ = 0.72, F = 26.29, p = 0.001, respectively; Fig 9).

**Table 6:** Correlation results between CO_2_ and CH_4_ fluxes of all the sectors of Chilika Lagoon.

Sector	n	r^2^	Equation
SS	16	72.1	FCH_4_ = 0.0008(FCO_2_)2–0.0091FCO_2_ + 0.0769
CS	12	67.7	y = 0.002 FCO_2_ + 0.02
NS	22	72	y = 0.001 FCO_2_ + 0.03

## Discussion

### CO_2_ dynamics in Seagrass, Macrophyte and Lagoon waters

#### Influence of forcing factors

The present study compares the role of coupled physical, chemical, and biological processes on the concentration of CO_2_ and subsequent emissions from seagrass meadows, macrophyte dominant sectors, and open lagoon waters. Both temporal and spatial changes in environmental and physical parameters influence biological processes, which in turn significantly modify the trace gas dynamics of the system. Robin et al. [44] reported several fold increase of fresh water inflow in the lagoon during the wet season. This resulted in a reduction in salinity coupled with increased SPM concentration during the wet season, which strongly influenced the seagrass-mediated CO_2_ dynamics and associated air-water exchange from the SS waters. Reduction of salinity by over 2.5 times at the SS from dry to wet season causes considerable physiological stress to the halophilic seagrass species. Substantial seasonal changes in water quality of the entire lagoon, particularly in seagrass dominated SS, possibly causes the observed reduction in the total seagrass area from 85 km^2^ to 65 km^2^ during the wet season [36]. This has also resulted in the release of excess CO_2_ due to enhanced decomposition of decayed seagrass and land derived organic detritus in the lagoon [56]. This condition also caused a proliferation of freshwater dominant macrophytes and algae, which resulted in an increase in CO_2_ emissions by 15 times at the SS (Table 2).

Similarly, higher pCO_2_ and corresponding higher CO_2_ flux (~ 9 times) during the wet season suggests the complex interactions of meteorological and physical forces (e.g. wind speed, freshwater inflow) and *in-situ* biological processes at the NS. Similar seasonal trend with highest community respiration during wet season, from various sectors of the lagoon was also reported [33, 57]. Despite the high rates of community respiration during the wet season [44, 58], we observed lower concentration of DIC at the NS as a result of its dilution due to increased freshwater inflow. Additionally, during the wet season there was a positive coupling between pCO_2_ and oxygen saturation in the NS, suggesting additional input of oxygen through the rivers. In contrast, limited seasonality was observed for pCO_2_ and CO_2_ fluxes from the CS waters. In addition to salinity stress, human induced stressors such as increase in turbidity through aquaculture, untreated domestic wastes, boating and fishing activities have led to a deterioration of seagrass health and its capacity to assimilate C [30].

#### Source-sink characteristics

DOC is considered as one of the major factors governing *in-situ* CO_2_ production through heterotrophic respiration [59]. Significant spatial variation in DOC during the wet season is a direct result of variable inflow of freshwater. Similar observations have also been reported from various estuarine [59] and lagoon systems [60, 61]. Although, pCO_2_ is inversely correlated to DOC, the shape of this relationship varied largely among sectors, suggesting large-scale sectoral differences in C delivery, quality, and *in-situ* processing as also reported by [62].

NS waters showed higher mean excess CO_2_ than the other sectors, although the DOC concentrations of both NS and SS waters were comparable. This perhaps is due to the marked differences in the nature of DOC, which has a higher labile fraction at the NS compared to the SS, while a non-labile fraction is prevalent at the SS. Negative excess DIC during dry season in SS clearly indicated a potential sink of atmospheric CO_2_ due to intense biological activities (e.g. high NCP) (Fig 4). In addition, reduced microbial activity, as observed by [44] on the non-labile fraction of DOC, resulted in lower concentrations of pCO_2_ at the SS during the dry season. Despite the substantial role of macroalgae in marine carbon sequestration and storage [63], abundance of labile material from the rivers supported higher microbial activity and increased pCO_2_ concentrations [44] in the macrophyte dominated NS. Higher pCO_2_ in this sector perhaps attributed to heterotrophic remineralization of organic detritus, the source of which is derived from both land-based inputs and *in-situ* degradation of macrophyte litter [64]. On the other hand, in SS, emission of CH_4_ dominated over CO_2_, clearly suggesting that CO_2_ is utilized for methanogenesis.

Positive values of net community production (NCP) in SS during both the seasons indicated net autotrophy during the annual cycle, probably due to the high primary productivity of benthic halophilic seagrass species. However, in NS, the observed NCP values indicated a seasonal shift in trophic state from heterotrophic (wet season) to the autotrophic (dry season). Similar seasonal heterotrophy was observed in other comparable freshwater dominated Indian systems such as Vembanad Lake [65]. Results indicate that both seasonality and vegetation play a key role in controlling source-sink dynamics of CO_2_ in Chilika lagoon. The macrophyte-dominant NS was a source of CO_2_ to the atmosphere through the year. The seagrass dominant SS was a sink of CO_2_ during the dry season and became a source during wet season when there was an obvious loss of seagrass. This clearly directs the role of vegetation in driving the source-sink dynamics of trace gases to the atmosphere.

### CH_4_ dynamics in seagrass, macrophytes and lagoon waters

CH_4_ concentration in the entire lagoon waters was observed to be high (>16 nM L^-1^) with the highest concentration in the seagrass dominated SS irrespective of seasonal changes. Larger wind speeds (4 m s^-1^) during the wet season resulted a two-fold increase in CH_4_ flux from the macrophyte-dominant NS, even though the concentrations were lower than that observed in the SS. Air-water gas flux has generally been considered to be wind independent under low-wind environments (e.g. <4 m s^−1^ [66]). However, higher wind speeds are known to be the primary source of surface turbulence with a potential to promote release of the dissolved gases to the atmosphere [67]. Significantly higher wind speed in NS (mean 4.04 ± 0.90 m s^-1^) during the wet season compared to the SS (mean 1.94 ± 0.65 m s^-1^) could explain the relative differences CH_4_ fluxes. The river waters entering to the lagoon were highly supersaturated (range 13.4–581 nM L^-1^) with CH_4_ and may have contributed significantly to the elevated CH_4_ concentration in the lagoon in the wet season. Similar values of high dissolved CH_4_ concentration (ranging between 4 and 573 nM L^-1^) have been reported in rivers and estuaries from the east and west coast of India [68]. Fig 5 shows conservative mixing of river water in the lagoon with high to moderate negative residuals of CH_4_ concentrations, which indicate the occurrence of possible outgassing and/or oxidation (e.g., [69], [70]) of CH_4_ in the lagoon during both the seasons. Among all sectors, macrophyte-dominated NS showed the highest negative CH_4_ residuals ranging from -66 to -160 nM L^-1^ (Fig 5). The results obtained for NS correlates with the results of Attermeyer et al [71] that indicated the potential role of floating macrophytes in reducing the emission flux of CO_2_ and CH_4_ from a tropical freshwater lake. In the SS residuals were lower, ranging from -83 to +35 nM L^-1^ during both seasons clearly indicating limited net oxidation/outgassing of CH_4_ from the system. The insignificant relationship between residual CH_4_ and salinity in SS further indicated the possible role of salinity independent *in-situ* processes in regulating CH_4_ concentration. Higher CH_4_ concentrations in SS are most likely the result of the active bubble ebullitions from the anoxic, organic carbon rich seagrass sediments, as observed by [72] for mangroves. Anilkumar et al [73] previously reported that the sediments of the lagoon are mostly mud (silt + clay), with an admixture of mud and fine sand at the southern and central sectors. In general, higher efflux of CH_4_ can be expected from fine sand/ silt-laden sediments, compared to other mud-dominant sediments [74] in the lagoon. This explains the higher CH_4_ efflux from the seagrass dominant SS.

Additionally, the mean salinity of the SS waters seldom exceeded a threshold value of 18 PSU, above which CH_4_ emission rates were significantly lower, compared to lower salinity levels [55]. Salinity driven inhibition of methanogenesis has been explained by the interspecies competition between the sulphate reducing bacteria [75, 76] and methanogens under high concentrations of SO_4_^2−^ in seawater. A distinct seasonality in seagrass densities was observed with higher densities (>60%) in summer due to dominant tidal influence and lower densities (<60%) in the wet season influenced by higher freshwater inputs. In fact, seagrass loss and decay are evident in the wet season, which contributes directly to enhanced CH_4_ fluxes in this study. Additionally, greater vascular plant cover is often linked with higher CH_4_ concentrations [77].

Among all the sectors, CH_4_ emissions relative to the CO_2_ emissions were the highest at the SS, which was largely attributed to photosynthetic CO_2_ uptake and possible carbon sequestration by seagrass meadows. The strong negative relationship between FCO_2_ and FCH_4_, indicated higher CH_4_ fluxes from the areas with low/negative CO_2_ fluxes (Fig 9). These observations resemble the relationship observed in a rice paddy [78]. The initial slope of this relationship was ~0.01mg CH_4_-C [mg CO_2_-C]^-1^, indicating that about 1% of recently fixed carbon was returned to the atmosphere as CH_4_ from the seagrass meadows. Additionally, there was a limited loss of biomass C via annual/seasonal harvesting unlike agricultural fields, which leads to the natural mineralization of the *in-situ* generated organic particles and slow turnover of sequestered carbon. This suggests that a large fraction of sedimentary CH_4_ escapes to the atmosphere, rather than being oxidized to CO_2_ through the water column in the SS. The higher residence time of CH_4_ in shallow SS waters resulted in reduced methane oxidation efficiencies leading to an “*epilimnetic shortcut*”. Previously, shallow waters have been identified as the hotspots for CH_4_ emission in lagoons and reservoirs due to limited CH_4_ oxidation rates in shallow water-column [79]. The positive relationship between FCO_2_ and FCH_4_ at the macrophyte-dominated NS and un-vegetated CS waters (Fig 9) could be due to the high influence of NS waters on CS.

The CH_4_/CO_2_ exchange stoichiometry estimated in the present study was mostly derived from measurements made during daytime when active photosynthetic CO_2_ uptake by the seagrass was most prevalent. Changes in the diurnal pattern of FCO_2_ and FCH_4_, especially when heterotrophic respiration dominates all over the meadows during night; and changes in the environmental parameters (e.g. salinity) may significantly alter the relative fluxes. Further, significant variations in GHG emissions between intra and inter meadows could also be attributed to the plant physiology and variations in sediment microbial communities [26]. This suggests the need for a more comprehensive year-round measurement of CO_2_ and CH_4_ flux considering the heterotrophic respiration at night to obtain a more accurate quantification of CH_4_/CO_2_ exchange stoichiometry.

## Conclusions

Positive air-water emission characteristics of CO_2_ and CH_4_ were observed from the Chilika lagoon, India’s largest brackish water lagoon. Prominent seasonality of CO_2_ fluxes was recorded at the seagrass dominant SS, while high emission rates of CO_2_ were characteristic of the macrophyte- dominant NS. The magnitude of CO_2_ efflux to the atmosphere from the SS waters was approximately eight times lower than the rest of the lagoon. It was further confirmed that the seagrass bed captures dissolved CO_2_ more effectively compared to other benthic ecosystems in the lagoon (e.g. macrophyte). The results demonstrated that a significant fraction of photosynthetically fixed carbon from the seagrass bed returned to the atmosphere as CH_4_.

CO_2_ emission was more dominant from the aquatic macrophyte-dominated NS predominantly due to high heterotrophic respiration compared to the other lagoon sectors. Excess organic inputs to the lagoon through anthropogenic supplies (such as domestic, agricultural wastes and industrial wastes from an upstream watershed area) could intensify the heterotrophic mineralization of anthropogenic detritus leading to enhanced GHG emissions from various redox conditions. The GHG source potentials further intensified due to land-based activities including agriculture, aquaculture and tourism reaching the NS from the surrounding villages. It can be summarized that the seagrass meadows in the Chilika lagoon act as a minor source of CH_4_ during both seasons and as minor sinks of CO_2_ during the dry season. However, in order to understand the CH_4_/CO_2_ exchange stoichiometry, it is necessary to obtain more comprehensive long-term measurements of CO_2_ and CH_4_ fluxes under varying environmental conditions.

## Supporting information

S1 Table Global Positioning System (GPS) (Trimble GOXR) locations of the sampling stations. (PDF)

S2 Table Seasonal variation of physico-chemical parameters, nutrients, sediment organic carbon, pCO_2_ and CH_4_ of surface waters of the Chilika Lagoon. (PDF)

S3 Table Seasonal variation of CO_2_ and CH_4_ concentrations and fluxes from surface waters of the Chilika Lagoon and rivers entering the Chilika Lagoon. (PDF)

S4 Table Influence of seagrass cover (%) on CH_4_ flux (mmol m^-2^ d^-1^) in Southern sector. (PDF)
