# Supplementary material for: Seagrass and macrophyte mediated CO2 and CH4 dynamics in shallow coastal waters
Source: PLoS One. 2018 Oct 8;13(10):e0203922. doi: 10.1371/journal.pone.0203922 (PMC6175284; doi:10.1371/journal.pone.0203922)
Supplement: S1 Table — (PDF) [file pone.0203922.s001.pdf]

Sample ID	Group	Latitude °N	Longitude °E
CH-S1	Southern sector - SS	19.534159	85.115964
CH-S2	Southern sector - SS	19.533047	85.151879
CH-S3	Southern sector - SS	19.533747	85.182994
CH-S4	Southern sector - SS	19.567432	85.182370
CH-S5	Southern sector - SS	19.600037	85.148784
CH-S6	Southern sector - SS	19.600039	85.182861
CH-S7	Southern sector - SS	19.603214	85.215946
CH-S8	Southern sector - SS	19.635574	85.181285
CH-S9	Southern sector - SS	19.636974	85.215688
CH-S10	Southern sector - SS	19.666796	85.181132
CH-S12	Southern sector - SS	19.667171	85.244052
CH-S17	Southern sector - SS	19.733234	85.250306
CH-S11	Central Sector - CS	19.665788	85.216641
CH-S13	Central Sector - CS	19.699000	85.214858
CH-S14	Central Sector - CS	19.699166	85.283810
CH-S15	Central Sector - CS	19.699101	85.348065
CH-S16	Central Sector - CS	19.698991	85.418582
CH-S18	Central Sector - CS	19.733784	85.318478
CH-S19	Central Sector - CS	19.733134	85.383794
CH-S20	Central Sector - CS	19.733093	85.449505
CH-S21	Northern Sector - NS	19.767054	85.283250
CH-S22	Northern Sector - NS	19.767657	85.349670
CH-S23	Northern Sector - NS	19.766942	85.416635
CH-S24	Northern Sector - NS	19.766657	85.483050
CH-S25	Northern Sector - NS	19.768411	85.544541
CH-S26	Northern Sector - NS	19.800980	85.383477
CH-S27	Northern Sector - NS	19.800016	85.450379
CH-S28	Northern Sector - NS	19.781408	85.516825
CH-S29	Northern Sector - NS	19.833574	85.417715
CH-S30	Northern Sector - NS	19.833242	85.485381
CH-S31	Northern Sector - NS	19.322107	85.499945
CH-S32	Outer Channel OC	19.667947	85.429935
CH-S33	Outer Channel OC	19.662751	85.463160
CH-S34	Outer Channel OC	19.664443	85.500496
CH-S35	Outer Channel OC	19.670154	85.520603
CH-R2	River to NS	19.906015	85.371765
CH-R3	River to NS	19.894279	85.400433
CH-R4	River to NS	19.918599	85.422543
CH-R5	River to NS	20.027550	85.512585
CH-R6	River to NS	19.971866	85.616641
CH-R7	River to NS	19.970442	85.621426
CH-R8	River to NS	19.948907	85.670205
CH-R9	River to NS	19.890770	85.816837
CH-R1	River to CS	19.604091	85.117037
CH-R13	River to CS	19.776862	85.227302
CH-R10	River to SS	19.727862	85.196111
CH-R11	River to SS	19.530536	85.090194
CH-R12	River to SS	19.656443	85.154388
