# Supplementary material for: Seagrass and macrophyte mediated CO2 and CH4 dynamics in shallow coastal waters
Source: PLoS One. 2018 Oct 8;13(10):e0203922. doi: 10.1371/journal.pone.0203922 (PMC6175284; doi:10.1371/journal.pone.0203922)
Supplement: S2 Table — (PDF) [file pone.0203922.s002.pdf]

Season	Sector	Sample ID	Water Temp.	Wind Speed	Salinity	pH (Probe)	DO Saturation	DIN	DIP	DSi	SPM	Chl a	DOC	DIC	SOC	pCO_2	CH_4	Excess DIC	Residual CH_4
	Units		*°C*	*m s^{-1}*	*psu*		*%*	*uM/L*	*uM/L*	*uM/L*	*mg/L*	*mg m^{-3}*	*mg/l*	*µmol/kg*	*% C*	*µatm*	*nM/L*	*µmol/kg*	
**DRY SEASON**	**Southern Sector -SS**	**CH-S1**	30.14	3.2	12.23	8.19	113.2	3.46	0.80	60.1	47.2	2.18	2.45	2330	0.79	576	12.8	59.9	-60.4
		**CH-S2**	30.38	3.5	13.60	8.20	112.7	3.32	0.83	64.6	114.7	3.12	2.41	1889	0.75	553	14.3	57.8	-71.5
		**CH-S3**	29.77	5.2	11.23	8.68	132.9	5.72	0.87	49.8	27.4	0.57	2.39	2553	1.46	548	73.8	-188.0	-64.7
		**CH-S4**	29.75	6.3	12.33	8.38	95.9	3.02	0.63	58.9	26.7	2.79	2.17	2183	1.32	335	55.1	-28.5	-77.5
		**CH-S5**	30.11	7.4	15.79	8.17	105.0	4.56	0.58	52.1	43.7	2.58	2.44	2197	0.69	521	14.4	45.4	-69.8
		**CH-S6**	30.56	6.5	15.41	8.21	118.2	5.25	0.61	48.3	41.2	4.63	1.93	2317	0.55	476	10.5	31.6	-45.7
		**CH-S7**	28.90	6.4	12.70	8.59	126.8	6.46	0.49	51.2	39.3	0.21	3.78	2251	1.25	180	50.9	-143.6	-79.7
		**CH-S8**	30.68	1.3	18.19	8.11	100.0	4.21	0.72	57.9	29.7	3.84	2.22	2244	0.70	585	17.8	65.4	-53.6
		**CH-S9**	28.94	1.7	14.17	8.53	119.6	7.53	0.51	49.2	33.7	0.34	2.72	2234	1.25	202	80.2	-119.8	-42.6
		**CH-S10**	30.34	1.9	18.39	8.02	102.8	3.09	0.72	55.0	91.7	5.44	2.85	2141	0.60	703	16.2	86.2	-64.2
		**CH-S12**	29.70	1.5	16.57	8.20	129.4	1.40	0.84	40.6	106.7	0.69	2.78	2226	0.72	464	35.1	26.8	-70.9
		**CH-S17**	31.21	2.3	20.64	8.11	120.1	4.35	0.80	49.2	40.7	4.40	2.42	2151	0.64	159	17.5	55.8	-50.8
	**Central Sector - CS**	**CH-S11**	30.55	0.5	17.89	7.96	120.0	3.94	0.93	89.9	64.2	7.48	2.08	2008	1.05	510	33.5	23.7	-69.5
		**CH-S13**	30.27	4.0	20.11	7.87	124.1	4.02	1.18	89.9	29.5	4.95	2.23	1992	0.74	1099	20.7	160.9	-70.5
		**CH-S14**	29.13	2.0	17.63	7.93	119.4	5.56	1.11	71.3	64.6	5.36	2.05	1866	0.71	587	18.8	36.7	-85.5
		**CH-S15**	29.16	3.0	19.22	8.10	95.6	3.11	1.18	18.6	46.0	6.75	2.34	1915	1.10	487	34.0	29.3	-61.9
		**CH-S16**	29.63	1.7	32.45	8.02	105.6	5.91	0.26	14.0	49.5	5.86	1.23	1730	0.11	605	15.1	75.9	-20.3
		**CH-S18**	29.23	3.4	23.71	7.95	110.7	2.27	1.00	66.7	119.3	4.43	1.83	1943	0.09	644	15.5	63.0	-56.5
		**CH-S19**	30.58	2.6	32.39	8.02	90.6	7.12	0.44	68.2	100.2	8.69	1.36	1841	0.67	687	22.2	113.2	-13.5
		**CH-S20**	31.24	1.1	23.61	7.98	118.9	2.97	1.00	55.8	158.4	6.36	1.85	1730	0.57	764	20.2	110.6	-52.3
	**Northern Sector - NS**	**CH-S21**	31.35	3.8	19.14	8.01	108.5	7.27	0.55	54.3	34.9	6.11	2.39	1816	0.88	678	26.2	79.0	-70.2
		**CH-S22**	30.48	4.1	25.40	7.98	69.5	7.43	0.38	35.7	71.8	7.31	1.95	1920	0.98	712	24.2	97.0	-72.1
		**CH-S23**	30.97	2.3	18.07	8.01	98.5	9.19	2.14	62.0	106.5	8.63	2.44	2050	0.32	774	19.5	107.7	-82.5
		**CH-S24**	30.88	1.7	17.17	7.95	114.6	8.05	0.38	46.5	72.8	8.92	2.64	2198	0.10	966	7.3	144.3	-99.5
		**CH-S25**	30.92	1.3	13.93	7.97	194.0	7.53	1.95	94.6	146.8	9.65	3.41	2301	1.14	1020	41.7	152.1	-82.4
		**CH-S26**	30.20	2.0	25.91	7.94	78.9	5.79	0.43	38.8	41.3	7.19	2.68	2197	0.66	889	20.7	149.0	-69.7
		**CH-S27**	30.61	1.9	14.16	7.92	101.9	5.59	0.51	62.0	155.8	9.30	3.40	2294	0.80	1160	15.0	165.6	-107.9
		**CH-S28**	30.60	1.4	16.82	8.03	142.8	5.01	0.91	138.0	36.4	14.25	2.51	1389	1.03	508	32.5	28.1	-76.2
		**CH-S29**	30.42	1.6	18.07	7.85	73.6	6.17	0.58	58.9	168.0	6.78	3.09	1581	0.21	880	15.4	88.9	-86.6
		**CH-S30**	30.68	1.9	7.51	7.65	83.4	7.28	0.43	86.8	132.3	12.78	3.12	1394	1.52	1518	77.6	92.7	-80.8
		**CH-S31**	30.43	2.4	15.83	8.10	128.6	6.35	0.67	111.6	27.1	5.52	2.83	2009	1.51	633	47.3	74.9	-66.7
	**Outer Channel - OC**	**CH-S32**	29.68	4.0	33.36	8.00	93.2	6.13	0.44	31.0	83.9	2.42	1.02	2026	0.55	674	23.6	102.6	
		**CH-S33**	31.26	4.2	33.67	8.07	119.6	3.79	0.37	21.7	78.9	3.18	1.05	2066	0.51	576	20.1	78.3	
		**CH-S34**	29.77	3.8	33.89	7.94	88.5	5.60	0.33	24.8	82.3	1.66	1.01	2147	0.24	826	16.6	146.3	
		**CH-S35**	30.19	4.2	33.91	7.96	84.0	4.03	0.41	17.1	27.7	2.46	0.84	2143	0.33	788	14.4	139.1	

		Sample ID	Water Temp.	Wind Speed	Salinity	pH (Probe)	DO Saturation	DIN	DIP	DSi	SPM	Chl a	DOC	DIC	SOC	pCO_2	CH_4	Excess DIC	Residual CH_4
		Units	*°C*	*m s^{-1}*	*psu*		*%*	*uM/L*	*uM/L*	*uM/L*	*mg/L*	*mg m^{-3}*	*mg/l*	*µmol/kg*	*% C*	*µatm*	*nM/L*	*µmol/kg*	
WET SEASON	Southern Sector -SS	**CH-S1**	28.2	3.00	7.85	8.01	96.4	7.58	0.58	74.6	74.0	2.56	4.02	1943	0.97	703	25.2	97.0	-9.3
		**CH-S2**	28.3	1.42	7.97	8.03	104.1	6.52	0.51	81.1	59.0	3.65	2.81	2071	1.22	543	47.8	81.9	2.5
		**CH-S3**	28.4	2.64	7.26	8.09	93.6	6.31	0.60	85.7	65.0	3.54	3.45	2013	1.00	647	71.8	75.0	-22.6
		**CH-S4**	28.8	1.72	7.49	8.17	112.4	7.11	0.40	47.4	57.0	4.76	3.58	1905	1.02	456	42.8	80.6	-33.9
		**CH-S5**	28.1	2.80	6.99	7.95	97.9	6.44	0.68	68.7	65.0	6.87	1.81	1946	0.90	774	49.6	112.9	-41.2
		**CH-S6**	28.5	1.31	6.94	7.93	104.2	5.84	0.68	70.8	64.0	6.66	1.98	1980	0.77	858	33.7	83.8	-16.8
		**CH-S7**	28.1	2.33	7.5	7.97	125.6	7.16	0.54	105.3	67.0	5.87	4.45	1809	1.35	750	48.4	106.4	-22.6
		**CH-S8**	27.7	1.20	5.84	8.05	100.0	7.19	0.38	75.7	44.0	2.98	2.92	1890	0.82	640	21.4	114.1	35.3
		**CH-S9**	27.8	1.50	5.99	8.04	102.0	7.75	0.23	98.4	130.0	1.89	4.03	1866	1.29	651	126.6	116.5	-74.4
		**CH-S10**	28.1	1.20	5.83	7.98	97.9	6.77	0.57	92.0	105.0	3.14	3.02	1914	0.79	721	19.7	119.5	-66.1
		**CH-S12**	26.3	2.36	5.18	7.95	98.9	5.86	0.53	75.1	134.0	2.88	2.33	1908	0.80	805	20.2	98.3	-83.1
		**CH-S17**	26.9	1.80	4.96	7.93	96.0	6.28	0.60	133.2	144.0	4.9	3.02	1921	0.77	821	16.0	66.9	-33.9
	Central Sector - CS	**CH-S11**	27.9	3.80	5.65	7.80	91.9	6.83	0.39	126.7	191.0	3.86	2.78	1186	0.71	1055	10.2	140.7	-87.0
		**CH-S13**	27.2	2.40	5.58	7.79	93.8	7.11	0.61	73.4	105.0	2.19	2.79	2136	1.01	1067	16.9	136.6	-81.5
		**CH-S14**	27.1	0.56	4.24	7.80	101.0	6.88	0.23	113.8	113.0	2.65	3.02	1274	1.06	982	11.9	117.5	-109.7
		**CH-S15**	27.8	2.47	3.73	7.74	94.5	7.79	3.38	113.3	165.0	2.87	3.10	1665	0.92	1198	12.7	137.3	-119.4
		**CH-S16**	27.9	4.10	1.08	7.64	89.1	6.24	0.29	147.3	145.0	3.56	3.06	1890	1.14	1424	35.4	129.8	-142.0
		**CH-S18**	27.7	4.64	4.37	7.85	96.8	9.74	0.97	133.0	116.0	5.32	3.02	1584	1.12	920	12.5	120.5	-109.7
		**CH-S19**	28.0	4.20	2.96	7.82	97.7	6.68	0.57	158.7	125.0	3.68	3.05	2130	1.05	956	19.2	113.5	-124.5
		**CH-S20**	25.9	5.31	0.55	7.56	95.0	7.25	0.13	152.1	280.0	2.11	2.30	1984	0.53	1690	9.1	140.4	-176.3
	Northern Sector - NS	**CH-S21**	27.8	3.06	3.37	7.46	88.0	8.93	0.84	82.9	102.6	4.69	5.12	2183	1.41	2664	17.3	237.1	-119.4
		**CH-S22**	28.0	2.70	5.22	7.39	96.9	8.11	0.90	135.5	102.6	5.62	4.22	2194	1.44	3025	10.1	263.8	-94.5
		**CH-S23**	26.5	2.96	0.31	7.39	94.4	7.56	1.54	167.0	351.0	5.77	4.06	1812	1.55	2637	28.7	195.8	-160.8
		**CH-S24**	25.9	4.30	0.54	7.37	96.9	8.64	0.98	171.4	313.2	6.85	3.86	1798	1.66	2723	43.4	197.9	-142.1
		**CH-S25**	26.2	3.79	1.48	7.40	102.0	10.78	0.59	119.0	224.1	6.83	4.05	2027	1.42	2864	27.3	225.1	-142.0
		**CH-S26**	26.1	3.83	3.95	7.49	97.1	8.24	0.57	108.3	306.9	5.52	3.64	1974	0.31	2145	10.8	191.4	-115.8
		**CH-S27**	26.3	3.55	1.94	7.44	87.6	7.91	0.79	139.3	277.2	6.21	3.26	1731	1.08	2241	29.4	170.3	-131.9
		**CH-S28**	26.3	3.77	0.13	7.29	94.7	11.61	1.35	161.6	339.3	8.10	4.21	1769	1.55	3228	47.4	219.9	-145.2
		**CH-S29**	25.5	5.54	0.52	7.41	90.3	7.54	0.57	124.2	142.2	5.21	4.88	1915	1.79	2653	53.5	203.3	-132.4
		**CH-S30**	25.9	5.68	1.15	7.33	89.9	10.66	0.54	148.4	117.0	4.84	3.45	1704	1.62	2857	49.1	196.5	-129.5
		**CH-S31**	27.4	4.12	0.51	7.28	91.9	5.71	1.21	161.1	41.4	4.24	4.49	1847	1.72	3469	50.8	237.6	-142.1
	Outer Channel - OC	**CH-S32**	27.5	2.10	7.99	7.75	89.4	5.36	0.33	160.5	113.0	2.23	1.50	1683	0.56	987	36.4	108.4	
		**CH-S33**	27.8	3.00	6.99	7.70	100.0	5.86	0.14	112.24	84.0	0.98	1.66	1668	0.89	1077	18.5	116.9	
		**CH-S34**	27.5	5.20	8.68	7.91	93.9	7.64	1.20	74.7	92.0	1.97	1.29	1766	0.62	676	15.8	91.7	
		**CH-S35**	27.9	5.20	16.49	7.99	94.4	6.87	1.06	99.1	106.0	1.34	1.20	1888	0.29	512	14.7	90.9	
