# Supplementary material for: Seagrass and macrophyte mediated CO2 and CH4 dynamics in shallow coastal waters
Source: PLoS One. 2018 Oct 8;13(10):e0203922. doi: 10.1371/journal.pone.0203922 (PMC6175284; doi:10.1371/journal.pone.0203922)
Supplement: S3 Table — (PDF) [file pone.0203922.s003.pdf]

		Units	*μatm*	*mmol m^{-2} d^{-1}*	*nM/L*	*mmol m^{-2} d^{-1}*	*mg CO_2-C m^{-2} d^{-1}*	*mg CH_4-C m^{-2} d^{-1}*
	**Sample ID**		***p*** **CO_2**	**CO_2 Flux**	**CH_4**	**CH_4 Flux**	**CO_2 Flux**	**CH_4 Flux**
**DRY SEASON**	**CH-S1**	**Southern Sector -SS**	576	16	12.8	0.042	4.30	0.03
	**CH-S2**		553	14	14.3	0.050	3.85	0.04
	**CH-S3**		548	9	73.8	0.391	2.59	0.07
	**CH-S4**		335	-10	55.1	0.344	-2.73	0.18
	**CH-S5**		521	21	14.4	0.094	5.75	0.07
	**CH-S6**		476	12	10.5	0.059	3.21	0.04
	**CH-S7**		180	-34	50.9	0.306	-9.24	0.23
	**CH-S8**		585	8	17.8	0.030	2.11	0.02
	**CH-S9**		202	-10	80.2	0.159	-2.77	0.12
	**CH-S10**		703	17	16.2	0.038	4.50	0.03
	**CH-S12**		464	3	35.1	0.064	0.82	0.05
	**CH-S17**		159	-31	17.5	0.047	-8.48	0.18
	**CH-S11**	**Central Sector - CS**	510	2.6	33.5	0.014	0.71	0.01
	**CH-S13**		1099	72.6	20.7	0.087	19.79	0.07
	**CH-S14**		587	10.4	18.8	0.039	2.84	0.03
	**CH-S15**		487	6.6	34.0	0.103	1.81	0.06
	**CH-S16**		605	10.6	15.1	0.067	2.88	0.05
	**CH-S18**		644	20.2	15.5	0.048	5.51	0.04
	**CH-S19**		687	18.3	22.2	0.060	5.00	0.05
	**CH-S20**		764	14.1	20.2	0.032	3.84	0.02
	**CH-S21**	**Northern Sector - NS**	678	26.8	26.2	0.110	7.30	0.08
	**CH-S22**		712	30.7	24.2	0.100	8.38	0.07
	**CH-S23**		774	24.0	19.5	0.051	6.54	0.04
	**CH-S24**		966	29.0	7.3	0.013	7.91	0.01
	**CH-S25**		1020	27.3	41.7	0.078	7.45	0.06
	**CH-S26**		889	27.0	20.7	0.047	7.36	0.03
	**CH-S27**		1160	43.3	15.0	0.033	11.80	0.02
	**CH-S28**		508	4.9	32.5	0.062	1.35	0.05
	**CH-S29**		880	24.0	15.4	0.030	6.54	0.02
	**CH-S30**		1518	66.7	77.6	0.099	18.20	0.07
	**CH-S31**		633	15.8	47.3	0.087	4.30	0.06
	**CH-S32**	**Outer Channel - OC**	674	24.6	23.6	0.067	6.70	0.05
	**CH-S33**		576	17.8	20.1	0.089	4.85	0.07
	**CH-S34**		826	36.6	16.6	0.047	9.99	0.03
	**CH-S35**		788	36.7	14.4	0.054	10.01	0.04

		Units	*µatm*	*mmol m^{-2} d^{-1}*	*nM/L*	*mmol m^{-2} d^{-1}*	*mg CO_2-C m^{-2} d^{-1}*	*mg CH_4-C m^{-2} d^{-1}*
	**Sample ID**		***p* CO_2**	**CO_2 Flux**	**CH_4**	**CH_4 Flux**	**CO_2 Flux**	**CH_4 Flux**
**WET SEASON**	**CH-S1**	**Southern Sector -SS**	703	25.0	25.19	0.075	6.82	0.06
	**CH-S2**		543	6.8	47.77	0.087	1.84	0.07
	**CH-S3**		647	18.6	71.84	0.209	5.07	0.08
	**CH-S4**		456	3.1	42.77	0.093	0.85	0.07
	**CH-S5**		774	29.8	49.61	0.148	8.11	0.08
	**CH-S6**		858	20.4	33.71	0.057	5.57	0.04
	**CH-S7**		750	25.8	48.38	0.136	7.04	0.09
	**CH-S8**		640	10.6	21.45	0.033	2.90	0.03
	**CH-S9**		651	13.2	126.59	0.257	3.60	0.12
	**CH-S10**		721	13.9	19.70	0.030	3.80	0.02
	**CH-S12**		805	28.6	20.23	0.046	7.81	0.03
	**CH-S17**		821	24.4	16.03	0.030	6.64	0.02
	**CH-S11**	**Central Sector - CS**	1055	66	10.20	0.032	18.13	0.02
	**CH-S13**		1067	48	16.93	0.040	13.05	0.03
	**CH-S14**		982	17	11.89	0.011	4.66	0.01
	**CH-S15**		1198	59	12.69	0.030	16.05	0.02
	**CH-S16**		1424	115	35.37	0.141	31.24	0.08
	**CH-S18**		920	64	12.51	0.049	17.47	0.04
	**CH-S19**		956	64	19.24	0.076	17.43	0.06
	**CH-S20**		1690	186	9.05	0.035	50.81	0.10
	**CH-S21**	**Northern Sector - NS**	2664	198	17.27	0.051	54.11	0.04
	**CH-S22**		3025	202	10.12	0.024	55.14	0.09
	**CH-S23**		2637	197	28.69	0.083	53.79	0.06
	**CH-S24**		2723	278	43.42	0.170	75.90	0.13
	**CH-S25**		2864	269	27.31	0.097	73.28	0.07
	**CH-S26**		2145	188	10.76	0.033	51.18	0.12
	**CH-S27**		2241	188	29.43	0.099	51.22	0.07
	**CH-S28**		3228	310	47.40	0.174	84.42	0.13
	**CH-S29**		2653	338	53.46	0.258	92.29	0.19
	**CH-S30**		2857	375	49.13	0.246	102.40	0.18
	**CH-S31**		3469	362	50.78	0.213	98.72	0.16
	**CH-S32**	**Outer Channel - OC**	987	36	36.36	0.081	9.93	0.06
	**CH-S33**		1077	60	18.47	0.056	16.26	0.04
	**CH-S34**		676	37	15.84	0.071	10.18	0.05
	**CH-S35**		512	14	14.72	0.090	3.95	0.07

	Sample ID		*p* CO_2	CO_2 Flux	CH_4	CH_4 Flux
	Units	*Units*	*µatm*	*mmol m^{-2} d^{-1}*	*nM/L*	*mmol m^{-2} d^{-1}*
**DRY SEASON**	CH-R2	**River to NS**	7099	501	452	1.55
	CH-R3		4283	394	231	1.08
	CH-R4		4911	366	362	1.28
	CH-R5		9062	648	232	0.80
	CH-R6		2907	151	163	0.45
	CH-R7		6128	294	356	0.89
	CH-R8		6845	435	581	1.81
	CH-R9		8529	630	39	0.19
	CH-R1	**River to CS**	2795	141	108	0.31
	CH-R13		9576	546	88	0.22
	CH-R10	**River to SS**	4363	402	84	0.3
	CH-R11		9149	925	22	0.1
	CH-R12		5863	294	471	1.2

		e ID	*p* CO_2	CO_2 Flux	CH_4	CH_4 Flux
		Units	*µatm*	*mmol m^{-2} d^{-1}*	*nM/L*	*mmol m^{-2} d^{-1}*
**WET SEASON**	CH-R2	**River to NS**	13833	1463	116	0.30
	CH-R3		11214	1023	107	0.38
	CH-R4		10368	613	176	0.50
	CH-R5		13406	1444	114	0.32
	CH-R6		9446	940	173	0.40
	CH-R7		4581	454	337	0.67
	CH-R8		13921	2781	217	0.54
	CH-R9		10763	687	13	0.03
	CH-R1	**River to CS**	8486	459	181	0.33
	CH-R13		8038	505	171	0.34
	CH-R10	**River to SS**	11464	488	205	0.62
	CH-R11		16596	651	233	0.80
	CH-R12		12723	583	319	0.56
