# Supplementary material for: Seagrass and macrophyte mediated CO2 and CH4 dynamics in shallow coastal waters
Source: PLoS One. 2018 Oct 8;13(10):e0203922. doi: 10.1371/journal.pone.0203922 (PMC6175284; doi:10.1371/journal.pone.0203922)
Supplement: S4 Table — (PDF) [file pone.0203922.s004.pdf]

	Dry Season		Wet Season	
Station	***Seagrass (% Cover)***	***FCH_4 (mM m^{-2} d^{-1})***	***Seagrass (% Cover)***	***FCH_4 (mM m^{-2} d^{-1})***
**CH-S1**	40	0.042	30	0.075
**CH-S2**	35	0.050	40	0.087
**CH-S3**	100	0.391	45	0.209
**CH-S4**	90	0.344	40	0.093
**CH-S5**	50	0.094	45	0.148
**CH-S6**	30	0.059	35	0.057
**CH-S7**	80	0.306	45	0.136
**CH-S8**	30	0.030	30	0.033
**CH-S9**	70	0.159	50	0.257
**CH-S10**	40	0.038	30	0.030
**CH-S12**	50	0.064	30	0.046
**CH-S17**	45	0.047	25	0.030
